# Accurate energies for ππ* excited states *via* exchange scaling: the XS-CASSCF method

**DOI:** 10.1039/d5sc09498d

**Published:** 2026-05-13

**Authors:** Rene F. K. Spada, Rodolpho L. R. Alves, Sayan Ghosh, Silmar A. do Monte, Lachlan Belcher, Ron Shepard, Hans Lischka, Felix Plasser

**Affiliations:** a Departamento de Física, Instituto Tecnológico de Aeronáutica São José dos Campos 12.228-900 SP Brazil rfkspada@ita.br; b Laboratório de Computação Científica Avançada e Modelamento (Lab-CCAM), Instituto Tecnológico de Aeronáutica São José dos Campos 12228-900 SP Brazil; c Departamento de Química, CCEN, Universidade Federal da Paraíba 58051-900 João Pessoa Brazil; d Department of Chemistry, Loughborough University Loughborough LE11 3TU UK f.plasser@lboro.ac.uk; e Chemical Sciences and Engineering Division, Argonne National Laboratory Lemont Illinois 60439 USA; f Department of Chemistry and Biochemistry, Texas Tech University Lubbock Texas 79409-1061 USA

## Abstract

The state-averaged complete-active space self-consistent field method (SA-CASSCF) is a widely employed electronic structure method used for studying photochemistry and dynamics owing to its ability to provide a reliable description even of complicated cases while still retaining computational efficiency. However, SA-CASSCF suffers from one Achilles heel, related to the description of ionic ππ* excited states, whose energy is often overestimated by 1–2 eV. In light of this challenge, we present the XS-CASSCF method, a new approach based on the idea of exchange scaling (XS) that screens the involved energy terms to improve the excitation energies of singlet ionic ππ* states. First, we illustrate the power of the XS-CASSCF method using hexatriene and *para*-quinodimethane as examples, showing that it corrects the targeted ionic states while leaving the other states largely unaffected, giving root-mean-square errors (RMSE) below 0.2 eV for the four lowest states in both cases. Subsequently, XS-CASSCF vertical excitation energies are tested against theoretical best estimates for a set of 11 molecules and 56 excited states. XS-CASSCF performs exceptionally well for the ππ* states of hydrocarbons, reducing the RMSE over 21 excitation energies from 0.96 to 0.27 eV. In the challenging subset of molecules with heteroatoms and a larger number of ππ* and nπ* states, we find that improvements can also be obtained, albeit not as pronounced. We conclude with an outlook into more realistic molecular materials focusing on their singlet–triplet (S_1_/T_1_) gaps, finding that significant improvements can be obtained along the whole range of S_1_/T_1_ gaps studied, going from 0.1 eV to more than 1.5 eV. Owing to notable improvements across significant classes of molecules combined with its conceptual simplicity, we believe that XS-CASSCF is a promising addition to the electronic structure toolbox, serving both as a standalone electronic structure method and as a starting point for further correlated treatment.

## Abstract 2

The XS-CASSCF method, a theoretically well-founded modification of the CASSCF method, significantly improves the excitation energies of singlet ionic ππ* states of a range of important chromophores.

## Graphical abstract

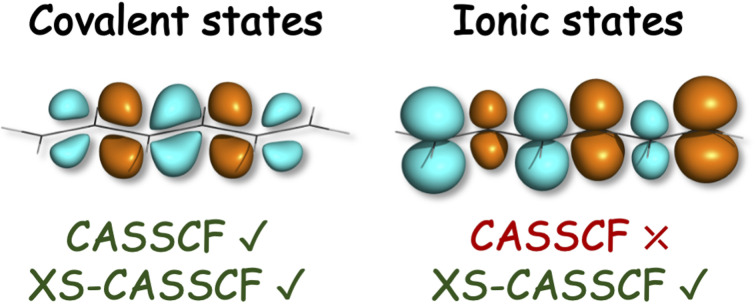

## 1 Introduction

Photochemical and photophysical processes play a crucial role in many areas of the molecular sciences. The study of these processes can drive the development of crucial technologies, such as the enhancement of solar cells *via* the singlet fission mechanism,^1–3^ development of new light emitters,^4–6^ and the creation of photonic devices for application in quantum technologies.^7,8^ Computational methods play an important role in this context by explaining experimental outcomes, providing insight into microscopic and ultrafast processes, and predicting new chemistry. However, the computational description of the underlying electronically excited states can be highly challenging^9^ and, therefore, the development of efficient and accurate computational methods is an on-going endeavor.^10–14^

Whereas standard single-reference methods including time-dependent density functional theory (TDDFT)^15^ are often beneficial for describing the bright excited states constituting the absorption spectrum, they can fail crucially for many interesting cases, such as the description of doubly excited states, strongly distorted molecular geometries, and non-radiative decay to the ground state.^9,16,17^ In such cases, a more general and flexible multireference framework is needed. Multireference computations usually start with a multiconfiguration self-consistent field (MCSCF) computation using often, specifically, the complete active space SCF (CASSCF) method.^18^ For excited states, the state-averaging (SA) formalism is often employed. SA-CASSCF can either be used as a computational method by itself or as the starting point for a further correlated treatment, *e.g.*, *via* multireference configuration interaction^19^ or CAS perturbation theory (CASPT2).^14^ The SA-CASSCF calculation plays a pivotal role in both cases. Clearly, no subsequent correlation treatment can fully eliminate the dependence on the CASSCF starting point. Crucially, if SA-CASSCF yields incorrect state ordering, then it is often very difficult to correct this problem in a later step leading to significant challenges.^20^

Notwithstanding its general power and flexibility, the SA-CASSCF method is plagued by one major Achilles heel. Indeed, it has long been known that SA-CASSCF, when used with standard valence active spaces, tends to severely overestimate the energies of the so-called “ionic” states,^21–25^ as understood within valence bond theory.^26,27^ Suggested solutions to describe ionic states require a significant increase in active space size^23,28^ or extensive inclusion of dynamic correlation.^24,29^ Both approaches imply a pronounced increase in computational cost and complexity, making them impractical for many molecules of interest.

It is interesting to compare the ionic states problem of SA-CASSCF with the much more well-known problem of TDDFT in treating charge-transfer (CT) states.^30^ The CT problem of TDDFT is by now well understood and CT states can be readily discovered using various popular diagnostics.^31–33^ Even more, the introduction of tuned range-separated hybrid functionals^34,35^ has led to an efficient general way of treating CT states within TDDFT using only one or two adjustable parameters. Taking inspiration from CT diagnostics for TDDFT, we have recently developed a diagnostic for ionic states^36^ in CASSCF, which indeed shows good correlation with computed SA-CASSCF errors. Here, we want to take one step further and aim to not only diagnose but also correct the problem.

An early attempt of correcting CASSCF revolved around simply globally scaling the excitation energies.^37^ A somewhat more targeted approach is the α-CASSCF method from Martínez and co-workers in which energy gaps to the state-averaged energy are scaled.^38^ Whereas these methods can be applied successfully in some cases, they are certainly only very rudimentary tools. For example, it would be impossible to correct errors in the ordering of the states in this way. As an alternative option, there are a number of methods combining MCSCF and DFT.^39–41^ These methods can produce accurate results, but their enhanced complexity may produce various formal and practical problems. For example, multiconfigurational pair density functional theory (MC-PDFT) is commonly used only as an *a posteriori* correction (retaining explicit dependence on the MCSCF starting point); a variational version of MC-PDFT has only been reported very recently^42^ and to the best of our knowledge gradients for state averaged MC-PDFT are not available at this time.

Within this work, we want to examine the question of what is the minimal required correction needed to obtain semi-quantitatively correct excitation energies of ionic states within CASSCF. Our aim is to directly modify the Hamiltonian in the iterative procedure, rather than changing the energies *a posteriori*, and thus go significantly beyond energy scaling approaches. Conversely, as opposed to CASSCF/DFT combinations, by keeping the correction to a minimum, such an approach will be more amenable to a variational wavefunction optimization, the computation of energy gradients and nonadiabatic couplings, and the implementation for other correlated methods.

We also take inspiration by the spin component scaled (SCS) and the scaled opposite-spin (SOS) *ab initio* methods, which consistently provide high-quality results for ground and excited-state properties generally providing improvements over the unscaled counterparts highlighting the possible benefits of targeted changes to *ab initio* methods.^43–45^ But as opposed to SCS and SOS approaches, we attempt to be even more targeted, scaling as little as possible.

We build our method on an observation made previously:^36,46^ the main energy term pushing up the ionic singlet excited states is the self-repulsion of the transition density between the ground and excited states. This self-repulsion, in turn, derives from the involved two-electron exchange integrals. Whereas in a fully correlated treatment these exchange integrals are screened *via* σ-correlation, we take a more pragmatic approach here and scale down the relevant integrals mimicking σ-correlation without additional computational cost. The new method is termed the exchange-scaled complete active space self-consistent field method (XS-CASSCF_[*µ*,*ν*]_). The method is based on two adjustable parameters, *µ* and *ν*, for scaling selected diagonal and off-diagonal Hamiltonian contributions, respectively. Along with XS-CASSCF, our implementation is also naturally extensible to other MCSCF expansion spaces, yielding a family of new XS-MCSCF methods.

This article is structured as follows: Section 2 presents the mathematical framework and working equations of the XS-CASSCF method. Section 3 describes the computational details. Section 4 presents results for hexatriene and *para*-quinodimethane (pQDM), illustrating the characterization of ionic and covalent excited states and the effect of exchange scaling, follows with a statistical analysis over a dataset of 11 molecules, and concludes with an investigation of the performance of XS-CASSCF for S_1_/T_1_ gaps of realistic molecular materials.

## 2 Methods

### 2.1 The MCSCF method

Considering the graphical unitary group approach (GUGA),^19,47–50^ the electronic Hamiltonian operator can be written in terms of the generators (*Ê*_*pq*_) of the unitary group as1
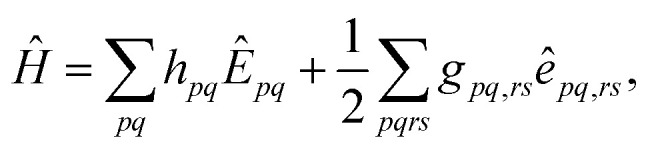
in which *h*_*pq*_ = 〈*p*|*h*|*q*〉 and *g*_*pq*,*rs*_ = 〈*p*(1)*r*(2)|*v*_12_|*q*(1)*s*(2)〉 are one- and two-electron integrals over the spatial orbitals. The unitary group generator is defined as

2 *Ê*_*pq*_ = *a*^†^_*p*α_*a*_*q*α_ + *a*^†^_*p*β_*a*_*q*β_

where *a*^†^_*p*α_ and *a*_*q*α_ are the creation and annihilation operators for MOs *p* and *q* with α-spin, respectively (and analogously for β-spin). The two-electron operator is written in terms of the unitary group generators as

3 *Ê*_*pq*,*rs*_ = *Ê*_*pq*_*Ê*_*rs*_ − *δ*_*qr*_*Ê*_*ps*_.

In the MCSCF method, the wavefunction is expanded in a basis of configuration state functions (CSFs) that depend on the MOs, and the CSF expansion terms determine the Hamiltonian matrix elements. Thus, in a truncated CSF basis, the MO coefficients affect the eigenvalues of the Hamiltonian operator. In the MCSCF procedure, the MO coefficients are optimized alongside the expansion coefficients of the CSF expansion to achieve a fully self-consistent approximation.

To reach reliable results for photochemical and photophysical applications, the excited states are optimized simultaneously with the ground state in the state averaging procedure and the same MOs and CSF expansion basis is employed for all considered states. Therefore, the CSF basis must be sufficiently flexible to describe simultaneously all the states of interest.

While the above discussion applies to any type of MCSCF expansion, we will focus below on the complete active space SCF (CASSCF) method. Within CASSCF all possible excitations within a chosen active orbital space are included in the wavefunction expansion.

### 2.2 Two-orbital two-electron model

We start the discussion by considering a two-orbital two-electron model using two spatial orbitals denoted *ϕ*_*p*_ and *ϕ*_*q*_. The wavefunctions for the singlet and triplet open-shell states, respectively |*Φ*^S^_*pq*_〉 and |*Φ*^T^_*pq*_〉, are given by a combination of two Slater determinants4
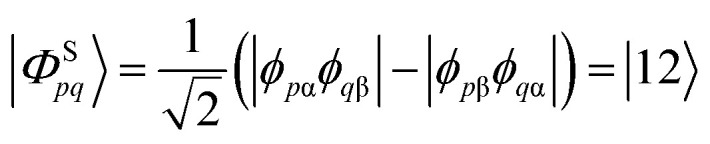
5
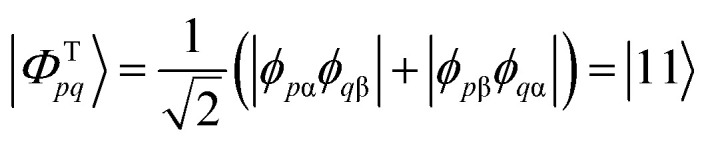
where the signs in the expansion determine the spin coupling. On the right-hand side of the above equations, we express the configurations using the GUGA step vector notation, where electronic configurations are represented as walks within the distinct row table (DRT). In this notation, “3” means doubly occupied, “1” means singly occupied and increasing the overall spin, “2” means singly occupied and decreasing the overall spin, and “0” means unoccupied.^18^

The energies associated with these states are^51,52^

6 *E*^S^_*pq*_ = *h*_*p*_ + *h*_*q*_ + *g*_*pp*,*qq*_ + *g*_*pq*,*qp*_,

7 *E*^T^_*pq*_ = *h*_*p*_ + *h*_*q*_ + *g*_*pp*,*qq*_ − *g*_*pq*,*qp*_,

in which *g*_*pp*,*qq*_ and *g*_*pq*,*qp*_ are the direct Coulomb and exchange terms, respectively. Combining these equations, we find that the singlet–triplet (S_1_/T_1_) energy gap between these states is given by the exchange term *g*_*pq*,*qp*_ as

8 Δ*E* = *E*^S^_*pq*_ − *E*^T^_*pq*_ = 2*g*_*pq*,*qp*_.

This exchange term can, in turn, be interpreted as the self-repulsion of the transition density,^46,52^ as will be illustrated below.

We note that the above discussion assumes that the molecular orbitals are identical for the singlet and triplet wave functions. The discussion becomes more complicated when orbital relaxation effects and correlation are taken into account.^52–55^ Nonetheless, we argue that the exchange interaction is the primary driving force in determining S_1_/T_1_ gaps, since it is the only explicitly spin-dependent term. From this perspective, orbital relaxation and correlation effects can be seen as secondary consequences of this interaction and will not be discussed further.

### 2.3 Ionic and covalent states

For the following discussion, it is beneficial to re-express the above equations in a localized orbital basis, which will ultimately clarify the meaning of “ionic” and “covalent” states (see also ref. 22, 27 and 36). Defining the localized orbitals *χ*_A_ and *χ*_B_ through9
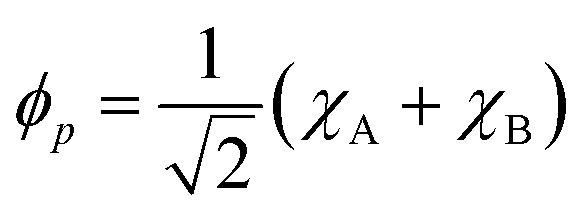
10
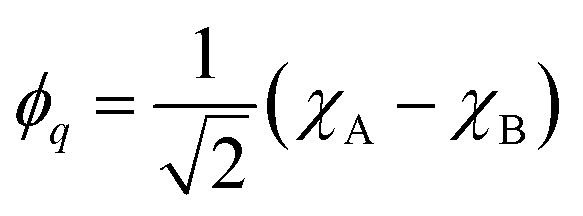


We can now express the singlet and triplet wave functions as11
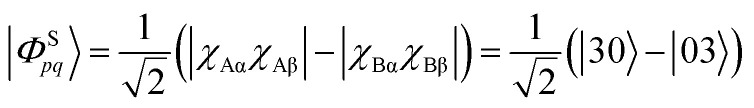
12
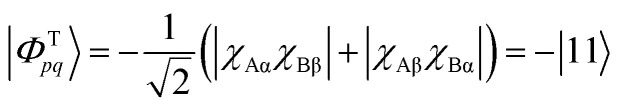


The crucial observation here is that the singlet state is composed of two configurations where the electrons are either simultaneously on *χ*_A_ or *χ*_B_, and these configurations are termed “ionic” or “zwitterionic”. By contrast, in the triplet state the two electrons are always on alternate sites, marking “covalent” or “diradical” character. As discussed in ref. 27, this type of construction not only applies to the presented two-orbital model system but is always possible for alternant conjugated hydrocarbons. Moreover, the classification into ionic and diradical states is also possible for more complicated cases where several electronic configurations are involved. To discuss such cases, one often applies Pariser's +/− nomenclature^56^ where singlet ionic states are characterized by a “+” sign and covalent states by a “−” sign.^27^

Eqn (11) explains why the singlet is higher in energy: the electrons are simultaneously on the same site, thus, experiencing enhanced Coulomb repulsion. Crucially, the dynamic nature of this state, with the electrons effectively hopping back and forth between the two sites, poses significant challenges for its computational description. Indeed, one finds that triplet states and covalent singlet states are usually described well by CASSCF. By contrast, singlet ionic state energies are often overestimated by more than 1 eV by CASSCF unless specialized large wave function expansions are used.

In a delocalized picture, following eqn (6), one finds that the enhanced interelectron repulsion is encoded within the exchange integral *g*_*pq*,*qp*_ (see also ref. 57). In a complete treatment, this enhanced repulsion is counterbalanced by σ-correlation. This σ-correlation is often so pronounced that it can be visualized as a characteristic contribution to the transition density,^36,46^ as exemplified below.

Rather than treating dynamic σ-correlation explicitly, we take a more pragmatic approach here. We argue that the combination of exchange repulsion and σ-correlation effectively produces a screened exchange interaction, which we incorporate as described below.

### 2.4 The XS-CASSCF_[*µ*,*ν*]_ method

The above discussion forms the basis for our strategy for defining a new method aiming to improve the energies for singlet ionic states. The main idea is that we want to mimic σ-correlation by screening the effective exchange interaction. To do so, we scale the exchange terms involved, and hence our method is denoted exchange-scaled (XS)-CASSCF. We first define a diagonal shift operator13
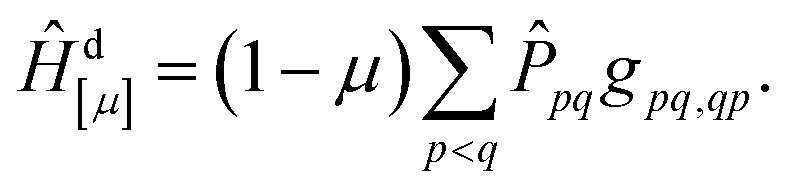
Here *P̂*_*pq*_ is defined as the projection operator into the space of all CSFs where orbitals *p* and *q* are singly occupied and singlet coupled while all other orbitals are either unoccupied or doubly occupied; it allows us to select the CSFs that we want to shift. Subtracting this shift Hamiltonian from the main Hamiltonian of eqn (1) would already provide a method where all singly excited singlet states are appropriately downshifted according to their associated exchange repulsion terms. However, we noticed that using only this diagonal shift provided an imbalance in the case of states where several configurations interact. We therefore also introduce an off-diagonal shift operator to reduce the splitting between CSFs that are shifted down in energy14
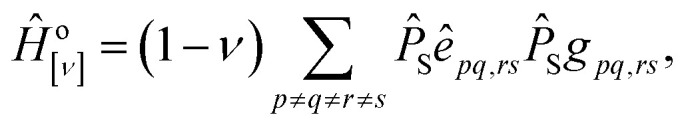
15
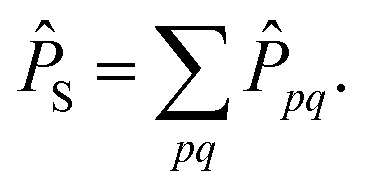
Here *P̂*_S_ is a projection operator into the space of all CSFs where any two orbitals are singly occupied and singlet coupled while all other orbitals are either unoccupied or doubly occupied.

Considering the diagonal and off-diagonal shift operators and the Hamiltonian operator in eqn (1), the scaled Hamiltonian becomes

16 *Ĥ*_[*µ*,*ν*]_ = *Ĥ*− *Ĥ*^d^_[*µ*]_ − *Ĥ*^o^_[*ν*]_,

in which the coefficients *µ* and *ν* are real numbers with values between 0 and 1 that introduce the scaling of the Hamiltonian terms. The influence of the shift operator is maximal if *µ* = *ν* = 0. Conversely, *µ* = *ν* = 1 means no shift and reproduces the standard MCSCF method.

Below, we exemplify the operation of the *P̂*_*pq*_ operator using various electronic configurations as expressed within the step vector notation

17 *P̂*_24_|31320〉 = |31320〉  *P̂*_24_|31023〉 = |31023〉

18 *P̂*_24_|33300〉 = 0  *P̂*_24_|11223〉 = 0

19 *P̂*_24_|12300〉 = 0  *P̂*_24_|31310〉 = 0

Considering the GUGA step vectors, the operator *P̂*_*pq*_, thus, has eigenvalues equal to one for the CSFs that have one, and only one, pair of singlet coupled singly occupied orbitals (*p* and *q*) and zero in any other case.

A different way to define the method is to state that we modify diagonal coupling coefficients as

20 〈*m*|*ê*_*pq*,*qp*_|*m*〉 ↦ *µ*〈*m*|*ê*_*pq*,*qp*_|*m*〉

whenever MOs *p* and *q* are singly occupied and singlet coupled with all other orbitals in the CSF |*m*〉 doubly occupied or unoccupied. Furthermore, we modify the off-diagonal coupling coefficients as

21 〈*m*|*ê*_*pq*,*rs*_|*n*〉 ↦ *ν*〈*m*|*ê*_*pq*,*rs*_|*n*〉

if and only if 〈*m*| and |*n*〉 were involved in the scaling according to eqn (20) and the indices *p*, *q*, *r*, *s* are all different from each other.

The *µ* and *ν* coefficients introduce controlled shifts that can fine-tune the electronic structure calculations for the singlet ionic ππ* states. We will discuss the selection of appropriate values for *µ* and *ν* below. In particular, we will investigate whether these have to be tuned in a molecule-specific way or whether we can find a set of universal parameters. Finally, although the shifted operator *Ĥ*_[*µ*,*ν*]_ affects only configurations with a single pair of singly occupied and singlet-coupled orbitals, small changes are expected for the energies of other configurations. This is because in the state-averaged MCSCF method all states are calculated simultaneously, and a change in *Ĥ*_[*µ*,*ν*]_ may influence the MO coefficients during optimization.

We note that the scaling procedure of eqn (20) and (21) does not only apply to the construction of the Hamiltonian matrix but also to the density matrices, and we implemented it for both. This means that the presented procedure is not just an energy correction, but is included in the MCSCF optimization process and can produce self-consistently optimized molecular orbitals, and consequently, wavefunctions. As such, the method is also naturally amenable to the computation of energy gradients and work to do so is currently in planning.

The XS-CASSCF method is implemented in a development version of the Columbus package,^58–61^ and is scheduled for release within Columbus version 7.3. Within Columbus, the *µ* and *ν* values are set using the xscale and xsoff keywords in the mcscfin file. Note that the XS-CASSCF procedure is turned on only for *µ* ≠ 1. As a technical note, XS-CASSCF is currently only implemented in the case where the Hamiltonian matrix is explicitly constructed and stored in memory (that is, setting npath = 11).

Within this work, we evaluate the scaling procedure as applied to the CASSCF approach yielding the XS-CASSCF method. Note, however, that the same procedure can be applied to any type of MCSCF expansion as available within Columbus yielding the more general XS-MCSCF method.

### 2.5 The *Q*^t^_a_ diagnostic for ionic states

Whereas the ionic state problem has been known for a long time,^21–24^ until recently, there has been no easy way to actually identify the problematic states. To this end, we have introduced the *Q*^t^_a_ diagnostic, which enables an automatic and quantitative detection of the ionic character of a state.^36^ We, furthermore, showed that the value of *Q*^t^_a_ is strongly correlated with the error in standard SA-CASSCF calculations^36^ providing a natural starting point for discussing the new energy correction.

As discussed in ref. 36, ionic states are associated with large transition charges on individual atoms, while these vanish for covalent states. In order to quantify the transition charge on atoms, we consider the Löwdin-orthogonalized one-electron transition density matrix *D̃*^t^ (1TDM), and obtain the transition charge on atom *M* as22
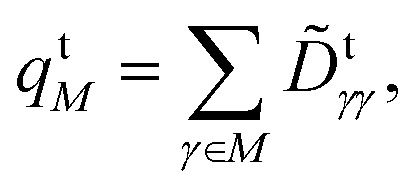
in which *D̃*^t^_*γγ*_ is a diagonal element of the 1TDM between the state of interest and the ground state; the index *γ* runs over all basis functions on atom *M*. The *Q*^t^_a_ diagnostic value can be derived from *q*^t^_*M*_ summing over the absolute values for each atom, that is,23
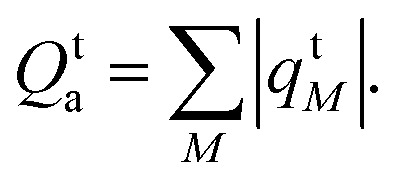
It is worth highlighting the use of the absolute value, as the direct summation of *q*^t^_*M*_ values yields zero whenever the states are orthogonal considering that also the overall integral over the transition density is zero.^62^

The *Q*^t^_a_ diagnostic is given in units of charge, and the values reported in this work are given in atomic units, that is, multiples of the unit charge *e*. In our previous work, we observed that for the case of ππ* states and using the Löwdin-orthogonalized 1TDM, values of *Q*^t^_a_ above 0.3*e* indicate that the state presents ionic character and is most probably not well described by standard SA-CASSCF.^36^

## 3 Computational details

Firstly, we selected the hexatriene and *para*-quinodimethane (pQDM) molecules for an in-depth analysis. For hexatriene, the molecular geometry was obtained from the QUESTDB database,^63^ which was optimized at the CC3/aug-cc-pVTZ level. Following this, we conducted both standard SA-CASSCF and the XS-CASSCF calculations with an active space of six electrons in six orbitals, CAS(6,6). These calculations included the ground state and five excited states, as specified in Table 1. We also performed multireference configuration interaction (MR-CISD) calculations^64^ using a (6,6) reference space applying the Pople extensivity correction.^65^ Note that the Pople correction applies only to the energies whereas the presented transition densities are equivalent to uncorrected MR-CISD.

**Table 1:** Excited states considered in the state-averaging procedure (along with the ground state) and active space for the SA-CASSCF and XS-CASSCF_[*µ*,*ν*]_ calculations

Molecule	Excited states	CAS
Ethene	^1^B_u_, ^3^B_u_	(2,2)
Butadiene	^1^A_g_, ^1^B_u_, ^3^A_g_, ^3^B_u_	(4,4)
Hexatriene	^1^A_g_, ^1^B_u_(2), ^3^A_g_, ^3^B_u_	(6,6)
Octatetraene	^1^A_g_, ^1^B_u_(2), ^3^A_g_, ^3^B_u_	(8,8)
Naphthalene	^1^B_3u_(2), ^1^B_2u_, ^3^B_3u_, ^3^B_2u_	(10,10)
Pyridine	^1^A_1_, ^1^B_1_, ^1^B_2_, ^1^A_2_, ^3^A_1_	(8,7)
Pyrimidine	^1^A_1_, ^1^B_1_(2), ^1^B_2_, ^1^A_2_(2), ^3^A_1_	(10,8)
Triazine	^1^A_1_, ^1^B_1_, ^1^B_2_, ^1^A_2_, ^3^A_1_	(12,9)
Acrolein	^1^A′(2), ^1^A″(2), ^3^A′, ^3^A″	(6,5)
Cyanoformaldehyde	^1^A″(2), ^3^A′, ^3^A″	(10,8)
Cyclopentadienone	^1^A_1_(2), ^1^B_1_, ^1^B_2_, ^1^A_2_, ^3^A_1_, ^3^B_1_, ^3^B_2_, ^3^A_2_	(8,7)

For the pQDM molecule, all the geometries were gathered from the previous work by Matasović,^57^ in which the planar structure was optimized with the PBE/ANO-S-VDZP methodology^66,67^ and then the CH_2_ groups were twisted up to 60°. For these calculations, the symmetry of the molecule was reduced to *C*_2h_, the SA-CASSCF and XS-CASSCF calculations were performed including 8 electrons in 8 orbitals (1a_g_, 3b_u_, 1a_u_, 3b_g_) in the active space. The considered states were 1^1^A_g_, 2^1^A_g_, 1^1^B_u_, 1^1^A_u_, 1^1^B_g_, 1^3^A_g_, 1^3^B_u_ and 1^3^B_g_. For both systems, the aug-cc-pVDZ^68^ basis set was considered.

**Fig. 1:**
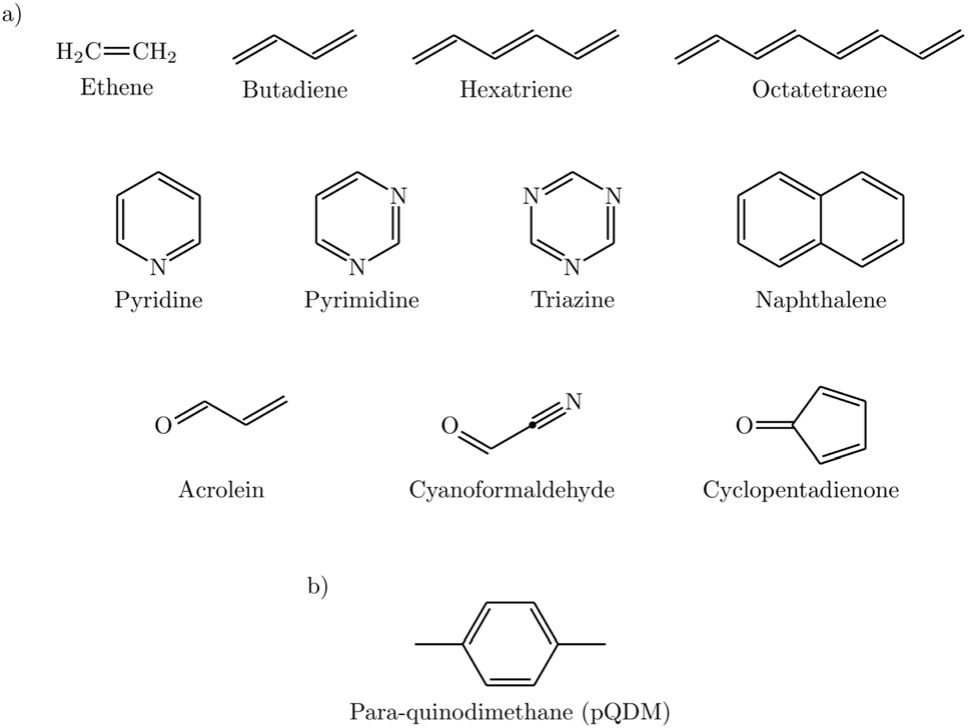
Molecular set considered in this work to apply the XS-CASSCF_[*µ*,*ν*]_ method: (a) molecules used for a statistical analysis of errors against QUESTDB; (b) the pQDM molecule studied individually.

Next, to assess the generality of the method, a set of 11 molecules was considered. The structures of the molecules are depicted in Fig. 1 and the geometries were taken from QUESTDB (optimized using CC3/aug-cc-pVTZ). At first, SA-CASSCF calculations were performed with equal weights for all states, as Table 1. Overall, 40 singlet states and 20 triplet excited states were calculated, and the excitation energies for 36 singlets and 20 triplets were used. Their values were compared with the QUESTDB theoretical best estimates (TBE) as computed using high-level coupled cluster or full CI depending on the molecule studied.^63^ The additional 4 singlet states were not available in QUESTDB but needed within SA-CASSCF in order to access the relevant states of interest, since the state ordering was altered between the two methods. All these calculations used the full molecular point group symmetry, except for ethene, in which the symmetry has been reduced to *C*_2h_.

XS-CASSCF was applied to the same set of molecules using the same parameters as standard CASSCF. We evaluated various *µ* and *ν* values in the range from 0.0 and 1.0 (see Fig. S1). From these data we calculated the root mean squared error (RMSE) and the signed mean error (ME) *vs.* the QUESTDB references. The aug-cc-pVDZ basis set was employed for all the SA-CASSCF and XS-CASSCF calculations.

In our analysis of the 11 molecules and the corresponding excited states listed in Table 1, we performed single-point calculations employing the time-dependent density functional theory (TDDFT) approach to obtain the excitation energies at this level. Utilizing the CAM-B3LYP functional, we preserved the same aug-cc-pVDZ basis set and molecular geometries as in the XS-CASSCF calculations. The TDDFT excitation energies were employed to carry out an error analysis, benchmarked against the reference values from QUESTDB. Subsequently, these errors were compared with those from the CASSCF and XS-CASSCF methodologies to assess comparative performance.

Further (XS)-CASSCF computations were performed on the molecules described in Section 4.4. The following levels of theory were employed, always with the cc-pVDZ^68^ basis set: XS-CASSCF(12,10)_[0,0.5]_ for DPP, O5P, and O6P; XS-CASSCF(8,8)_[0,0.5]_ for diBN and DiKTa; XS-CASSCF(8,8)_[0,1]_ for pentacene; XS-CASSCF(10,10)_[0,0.5]_ for mDICz; XS-CASSCF(10,10)_[0,1]_ for CzBN. On this set of molecules we also performed spin-component scaling second-order approximate coupled-cluster (SCS-CC2)^45,69^ calculations with the def2-TZVP^70^ basis set using the Turbomole 7.4 (ref. 71) program system, employing the resolution of identity (RI) approximation and freezing core electrons from the correlation treatment. Additionally, TDDFT calculations were conducted using the CAM-B3LYP^34^ functional and the def2-TZVP^70^ basis set. These generally used the full RPA-TDDFT formalism;^72^ only in the case of pentacene, we applied the Tamm-Dancoff approximation^73^ due to numerical problems. The TDDFT computations were carried out using the Q-Chem 6.3 (ref. 74) software package.

All (XS)-CASSCF and MR-CI calculations were performed using a development version of the Columbus package.^19,58–61^ The *Q*^t^_a_ diagnostic was computed for the singlet states using the TheoDORE 3.1.1 program package.^75^

## 4 Results and discussion

### 4.1 Hexatriene

**Fig. 2:**
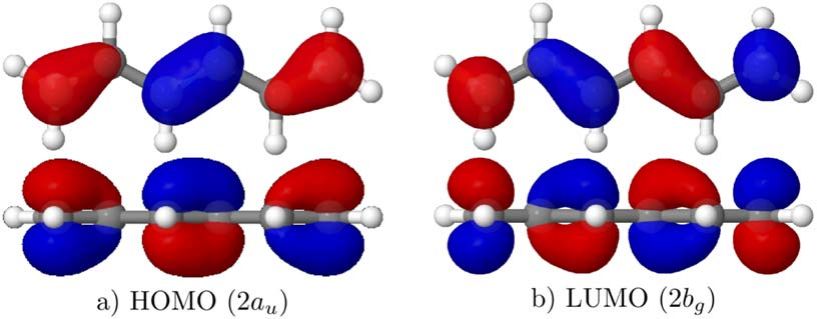
Frontier MOs of hexatriene: (a) HOMO and (b) LUMO.

We start the discussion by illustrating the main properties of the XS-CASSCF method using the example of hexatriene. Its highest occupied (HOMO, 2a_u_) and lowest unoccupied molecular orbitals (LUMO, 2b_g_) are shown in Fig. 2. The first singlet excited state (1^1^B_u_^+^) arises from a HOMO/LUMO single excitation. This is followed by the 2^1^A_g_^−^ state formed from the HOMO/LUMO double excitation along with further contributions from the HOMO−1 and LUMO+1. The first triplet state (1^3^B_u_^−^) possesses a configuration analogous to 1^1^B_u_^+^. All of these are ππ* states.

**Fig. 3:**
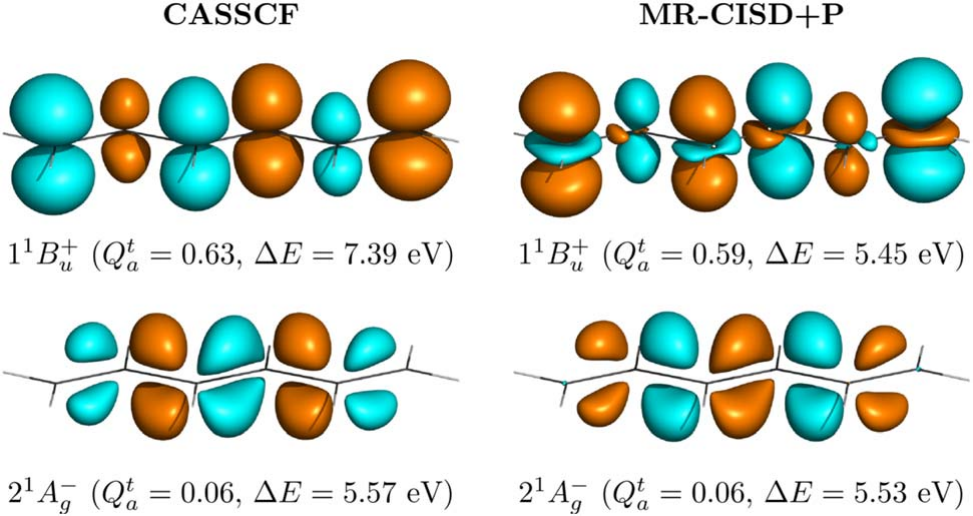
Transition densities for the 1^1^B_u_^+^ (top) and 2^1^A_g_^−^ (bottom) states of hexatriene computed at the CASSCF and MR-CISD+P levels. *Q*^t^_a_ diagnostic values and excitation energies are given in parentheses.

To illustrate the character of the two lowest singlet states, we will investigate their transition densities. Starting with CASSCF, as shown on the left in Fig. 3, we find that for the 1^1^B_u_^+^ state the transition density is centered near the atoms occupying individual p-orbitals.^27,36^ By contrast, the transition density for the 2^1^A_g_^−^ state is located around the bonds, arising from the overlap of adjacent p-orbitals. In line with previous discussions, the former marks ionic character whereas the latter indicates covalent character.^27,36^ While this visual analysis is a good starting point, it is beneficial to have a more immediate description. For this purpose, we developed the *Q*^t^_a_ diagnostic^36^ where *Q*^t^_a_ values above around 0.3 indicate ionic character. The *Q*^t^_a_ values obtained from CAS(6,6)/aug-cc-pVDZ calculations are also presented in Fig. 3. The strong difference between these values (0.63 *vs.* 0.06) underscores the difference in state character.

Moving to the MR-CISD transition densities shown on the right in Fig. 3, we find that these are very similar in appearance to the CASSCF transition densities. However, for the 1^1^B_u_^+^ state, additional σ-contributions become visible. The excitation energies for CASSCF and MR-CISD+P are nearly identical for the 2^1^A_g_^−^ state (5.57 *vs.* 5.53 eV), whereas the 1^1^B_u_^+^ state shows a significant 2 eV decrease (7.39 *vs.* 5.45 eV).

To explain the above observations, it is first worth noting that the transition density self-repulsion can be shown to contribute to the singlet excitation energy, in analogy to how the Hartree term (the density self-repulsion) contributes to the ground state energy.^46^ Thus, enhanced transition density self-repulsion means an increased excitation energy. The term is higher for the ionic 1^1^B_u_^+^ state where the transition density resides directly in the p-orbitals above the atoms compared to the covalent 2^1^A_g_^−^ state where overlap distributions are involved. This also explains why the HOMO/LUMO 1^1^B_u_^+^ state is significantly higher in energy than the 2^1^A_g_^−^ state, despite the latter involving higher energy orbitals and double excitations. At the MR-CISD+P level, the energy of the 1^1^B_u_^+^ state is lowered due to the admixture of σ → σ* single excitations. In the transition density these become visible as opposite sign σ-contributions effectively lowering the transition density self-repulsion.^46^

The excitation energies for hexatriene are listed in Table 2 comparing standard SA-CASSCF(6,6), XS-CASSCF(6,6), MR-CISD+P and the theoretical best estimates (TBE) from QUESTDB. Comparing, first, standard SA-CASSCF with TBE, we note that the two methods agree extremely well (within 0.05 eV) for the covalent 2^1^A_g_^−^ state and for both triplet states. By contrast, the energy of the ionic HOMO/LUMO (1^1^Bu^+^) state is strongly overestimated, with an error of approximately 2 eV. Even more, there is a stark discrepancy of the singlet-state ordering. The 1^1^B_u_^+^ state is the lowest singlet state (at 5.37 eV) within the reference computations but it is the third singlet (at 7.39 eV) for standard SA-CASSCF. There is also a notable discrepancy in the S_1_/T_1_ gap, which is noteworthy in view of hexatriene as a model singlet fission chromophore:^76^ while the TBE for the S_1_/T_1_ gap is 1.01 eV, this value increases three-fold to 3.08 eV when using standard SA-CASSCF.

**Table 2:** Excitation energies for the hexatriene molecule calculated with standard SA-CASSCF (CAS) and XS-CASSCF (XS-CAS) using [*µ*,*ν*] = [0,0] and [0,0.5], all based on a (6,6) active space, compared to MR-CISD+P (MRCI) and the theoretical best estimates (TBE)

State	CAS	XS-CAS [0,0]	XS-CAS [0,0.5]	MRCI	TBE^a^
2^1^A_g_^−^	5.57	5.61	5.34	5.53	5.62
1^1^B_u_^+^	7.39	5.32	5.59	5.45	5.37
1^1^B_u_^−^	6.79	8.49	7.07	6.81	
1^3^A_g_^+^	4.31	4.32	4.33	4.42	4.36
1^3^B_u_^−^	2.72	2.72	2.73	2.78	2.73

a TBE from ref. 63.

As illustrated in Fig. 3, the ionic 1^1^Bu^+^ state is destabilized by the self-repulsion of the transition density which, in turn, can be approximated as the exchange integral involving the 2a_u_ (HOMO) and 2b_g_ (LUMO) orbitals. In a general CI calculation, the influence of this term would be counter-balanced by σ-correlation. Indeed, using a correlated multireference treatment, as shown above in the case of MR-CISD+P, it is possible to lower the 1^1^Bu^+^ state to its appropriate energetic position, albeit at the cost of significantly increased computational effort (see also ref. 29).

We will now evaluate the effectiveness of the XS-CASSCF approach, which emulates this effect by scaling the exchange integrals involved, not requiring explicit treatment of the σ-electrons. Starting with XS-CASSCF_[0,0]_, that is, completely neglecting the exchange terms and the related off-diagonal coupling terms, we obtain excellent results and the error for 1^1^B_u_^+^ is reduced from 2.02 to 0.04 eV. Crucially, the three other low energy states are not affected and we are now able to describe the four lowest energy states all within 0.05 eV of the TBE reference thereby also restoring the correct state ordering.

Viewing Table 2 in more detail, we find that despite providing a perfect description of the first four states, XS-CASSCF_[0,0]_ significantly pushes up the 1^1^B_u_^−^ state placing it at 8.49 eV. No QUESTDB reference for 1^1^B_u_^−^ is available but our MR-CISD+P calculations show that this state should be at 6.81 eV in line with the original unscaled SA-CASSCF results. If an accurate description of this state is required as well, then we have to adjust the off-diagonal scaling *ν*. Setting *ν* = 0.0 works well for the low-energy states but pushes the 1^1^B_u_^−^ state too high up in energy. The reason is that this state is formed as a linear combination of the HOMO−1/LUMO and HOMO/LUMO+1 transitions and, therefore, sensitively depends on the coupling between these configuration which, in turn, is modulated by *ν*. Viewing now the results from XS-CASSCF_[0,0.5]_, we find that this method is able to reproduce all five states shown within an accuracy of 0.3 eV. At the same time, we note that the description of 2^1^A_g_^−^ and 1^1^B_u_^+^ is slightly less accurate.

**Fig. 4:**
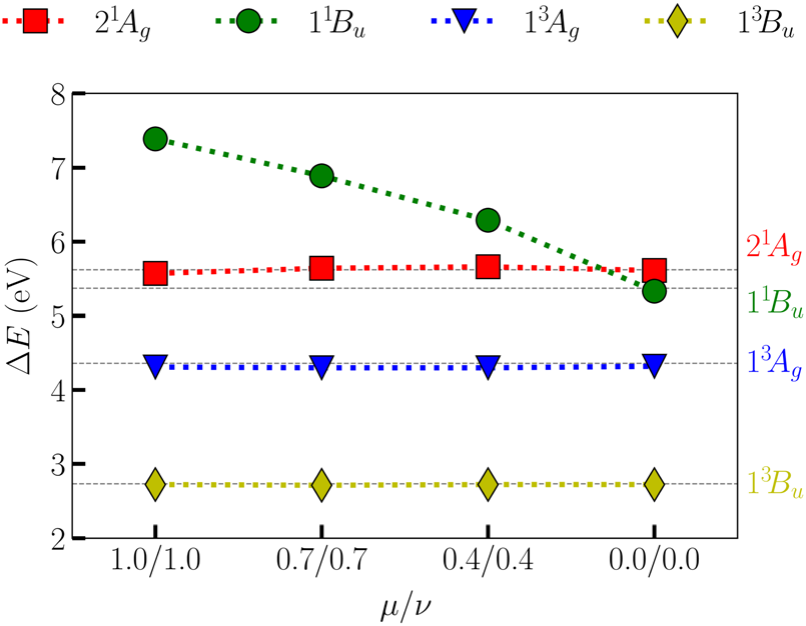
Vertical excitation energies (Δ*E*) for the 2^1^A_g_, 1^1^B_u_, 1^3^A_g_ and 1^3^B_u_ states of the hexatriene molecule. The dashed horizontal lines represent the vertical excitation energies gathered from QUESTDB.

To investigate the evolution of the vertical excitation energies, we performed further XS-CASSCF_[*µ*,*ν*]_ calculations considering [*µ*,*ν*] values of [1.0,1.0], [0.7,0.7], [0.4,0.4] and [0.0,0.0]. These results are shown in Fig. 4, and from the analysis of the data, one can see that XS-CASSCF can significantly reduce the error values for the ionic state (1^1^B_u_^+^) while keeping the good concordance of the excitation energies for the other low-energy states. Notably, there is a steady decrease of 1^1^B_u_^+^ whereas the other states remain constant throughout. As discussed above, if a larger number of states, including 1^1^B_u_^−^, are of interest, then the combination [0.0,0.5] is recommended.

### 4.2 *Para*-quinodimethane (pQDM)

As a more challenging case to test the capabilities of the XS-CASSCF method in the presence of multiple states, we considered the pQDM molecule following recent work by some of us.^57,77^ The calculations were performed considering a CAS(8,8) averaging over five singlet states and three triplet states.

**Fig. 5:**
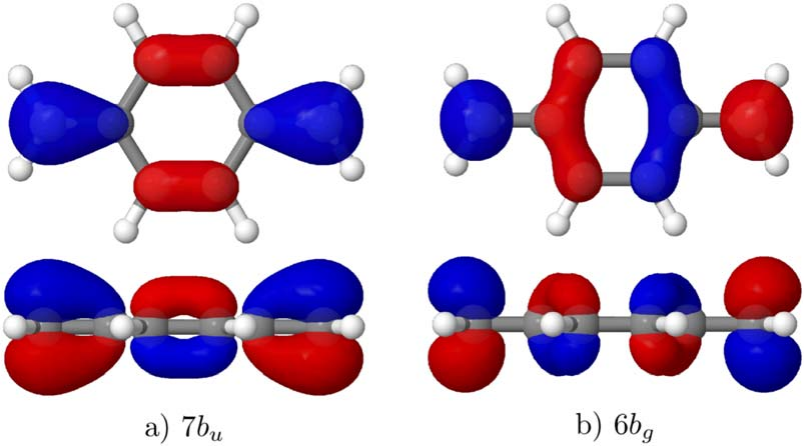
Frontier orbitals of pQDM: (a) HOMO (7b_u_) and (b) LUMO (6b_g_).

The relevant orbitals for the following discussion are the 7b_u_ (HOMO) and 6b_g_ (LUMO), as shown in Fig. 5. The ground state (1^1^A_g_) represents the closed shell configuration. The 2^1^A_g_ state has strong contributions from the 7b_u_ → 6b_g_ double excitation. The bright 1^1^A_u_ state along with the 1^3^A_u_ state are formed by a 7b_u_ → 6b_g_ single excitation. The other states involve orbitals aside from HOMO and LUMO.

The vertical excitation energies obtained for standard SA-CASSCF(8,8), XS-CASSCF(8,8)_[0,0]_, and MS-CASPT2 are found in Table 3. In addition, we present the *Q*^t^_a_ diagnostics, computed at the SA-CASSCF(8,8) level for the singlet states. The discussion will focus on the A_g_ and A_u_ states; the B_g_ and B_u_ states are shown for completeness. We find a similar pattern as before: the covalent 2^1^A_g_ state and the triplet states are well-described. By contrast, the 1^1^A_u_ state is strongly overestimated with an energy of 6.82 eV *vs.* the reference of 4.78 eV. As a consequence, 1^1^A_u_ now becomes S_4_ at SA-CASSCF whereas it is S_1_ for MS-CASPT2, thus, completely shifting the state ordering. Utilizing the XS-CASSCF_[0,0]_ approach, we observe a stabilization of the 1^1^A_u_ electronic state putting it at 4.74 eV very close to the CASPT2 reference and correctly placing it within ±0.1 eV of the 2^1^A_g_ state. As before, XS-CASSCF_[0,0]_ leaves the other states largely unaffected with 1^1^B_u_ being the only state that is shifted by more than 0.3 eV.

**Table 3:** Excitation energies for the pQDM molecule calculated with standard SA-CASSCF(8,8), XS-CASSCF(8,8)_[0,0]_ and MS-CASPT2; *Q*^t^_a_ values are presented for SA-CASSCF(8,8)

State	CAS(8,8)	*Q* ^t^_a_	XS-CAS(8,8)_[0,0]_	MS-CASPT2^a^
2^1^A_g_	4.87	0.135	4.64	4.89
1^1^B_u_	6.68	0.038	7.34	6.77
1^1^A_u_	6.82	0.334	4.74	4.78
1^1^B_g_	5.65	0.034	5.34	4.68
1^3^A_g_	4.43		4.46	4.71
1^3^A_u_	2.19		2.22	2.28
1^3^B_g_	4.00		4.03	4.16

a Results from ref. 57 using an IPEA shift of 0.25.

**Fig. 6:**
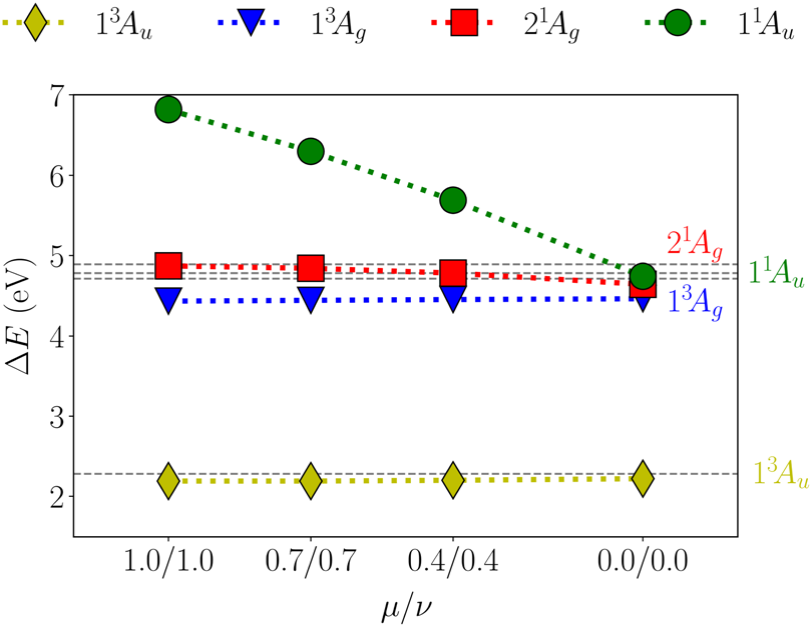
Vertical excitation energies (Δ*E*) for the pQDM molecule considering the lowest singlet and triplet states of A_u_ and A_g_ symmetry. The dashed lines refer to MS-CASPT2 reference values from ref. 57.

Variations of the *µ* and *ν* parameters are investigated in Fig. 6 (see also Fig. S2). As discussed above, for the standard SA-CASSCF calculation, the ionic 1^1^A_u_ state is the fourth excited state lying at 6.82 eV. Decreasing *µ* and *ν* we find a smooth decrease of the 1^1^A_u_ state to its expected value leaving the other states largely unaffected. Finally, setting *µ* and *ν* both to 0.0 puts the 1^1^A_u_ state close to the 1^3^A_g_ and 2^1^A_g_ states, with an excitation energy of 4.74 eV. The proximity of these states is in line with the prediction by the MS-CASPT2 calculation shown as the dashed lines in the plot. It is also worth mentioning that the triplet states are not affected by the XS-CASSCF approach, keeping an almost constant energy for all values of *µ* and *ν*.

**Fig. 7:**
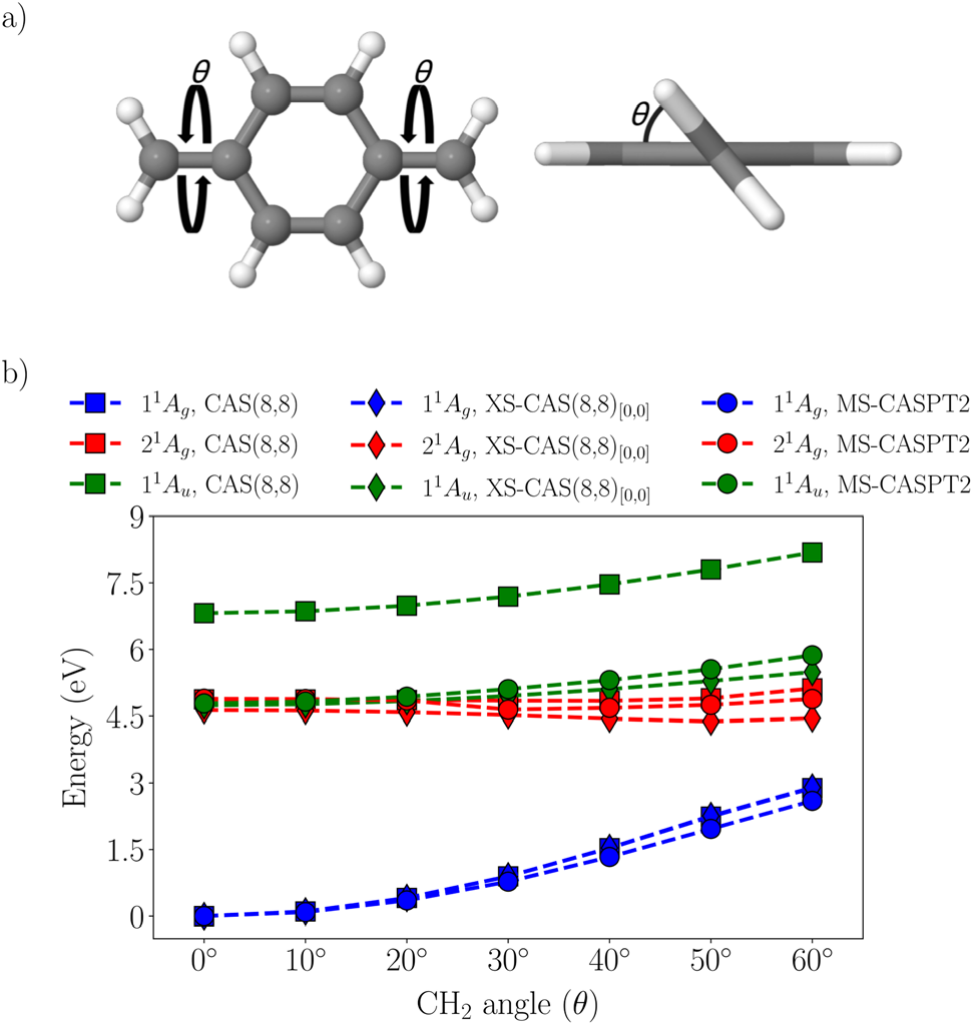
Analysis of twisted pQDM: (a) definition of the twisting angle *θ*, (b) potential energy curves for standard SA-CASSCF(8,8), XS-CASSCF(8,8)_[0,0]_, and MS-CASPT2.

Next, we were interested in how XS-CASSCF performs in the case of twisted pQDM geometries. Following ref. 57, geometries were constructed by varying the torsional angle *θ* between the π-plane and the CH_2_ groups, as illustrated in Fig. 7a. This variation allows for the investigation of how structural distortions influence the electronic states and their corresponding excitation energies, providing a deeper understanding of the molecular response to geometric changes. Specifically, we were interested in investigating whether the superior performance of XS-CASSCF also holds as the ground state obtains enhanced open-shell character.

Starting with the MS-CASPT2 results (circles in Fig. 7b), we find in analogy to Table 3 that at the planar geometry the 2^1^A_g_ and 1^1^A_u_ states are quasi-degenerate at a vertical excitation energy of 4.8 eV. The most prominent change upon twisting is a strong increase of the ground state energy (blue circles in Fig. 7b), related to breaking of the π-bonds, and at a twisting angle of *θ* = 60°, the ground state lies at 2.60 eV. The 2^1^A_g_ state (red circle) remains almost unchanged while the 1^1^A_u_ state increases slightly in energy. Viewing standard SA-CASSCF, we find that the 1^1^A_g_ and 2^1^A_g_ states are reproduced almost perfectly well, *i.e.*, the blue and red squares lie almost on top of the blue and red circles. By contrast, the 1^1^A_u_ state is consistently severely overestimated (by about 2 eV). Such a strong overestimation is certainly in no way an acceptable starting point for dynamics simulations or further explorations of the potential energy surface.

Noting the unsatisfactory performance of standard SA-CASSCF, we were interested in whether XS-CASSCF would perform better. The XS-CASSCF_[0,0]_ results are presented as diamonds in Fig. 7b. We find that the description of the 1^1^A_u_ state is greatly improved while the energies of the other two states are almost unaltered. Viewing Fig. 7b, we find that the XS-CASSCF_[0,0]_ diamonds are closely aligned with the MS-CASPT2 circles and that this is true for all states and geometries considered. Crucially, we find that the performance of XS-CASSCF does not deteriorate for the twisted geometry and we are, thus, hopeful that XS-CASSCF will not only be useful for vertical excitation energies but will also present itself as a powerful method for the exploration of potential energy surfaces and dynamics.

### 4.3 Dataset

Encouraged by the good performance on hexatriene and pQDM, we now assess the general applicability of the XS-CASSCF method using a set of 11 molecules, as presented in Fig. 1, investigating 56 excited states. This same set was employed in our previous work,^36^ comprising a diverse set of nπ* states, and covalent and ionic ππ* states, considering singlet and triplet spin multiplicities. We performed XS-CASSCF_[*µ*,*ν*]_ calculations varying the *µ* and *ν* parameters as illustrated in Fig. S1 and computed RMSE and ME with respect to the reference values. The full results using all tested parameter combinations can be found in the supplementary information (Tables S1 to S13 and Fig. S3 to S15) and we will focus on the optimised parameter combinations here.

Within the following, we will investigate two different options for determining the *µ* and *ν* parameters. On the one hand, we will use the globally optimised parameter combination [*µ*,*ν*] = [0.0,0.5], which produced the lowest overall RMSE over all molecules. On the other hand, we will apply tuned molecule-specific parameters. We will discuss which approach will be more suitable in practice.

First we carried out an analysis of the results for the five hydrocarbons, that is, ethene, butadiene, hexatriene, octatetraene and naphtalene. The results for standard SA-CASSCF as well as XS-CASSCF using globally and individually optimised parameters are listed in Table 4. Starting with standard SA-CASSCF, we note the substantial deviations from the reference values. The individual RMSEs for the molecules are around 1 eV; the overall RMSE is 0.96 eV. Applying, first, the globally optimized XS-CASSCF_[0.0,0.5]_ method already substantially improves the excitation energies for all five hydrocarbons. The largest improvement is for hexatriene, in which the RMSE decreases from 1.01 to 0.18 eV. The RMSE value over all molecules is lowered to about a third of the original value from 0.96 to 0.38 eV.

**Table 4:** RMSE values (eV) for the hydrocarbon test set calculated using standard SA-CASSCF, XS-CASSCF with globally and individually optimized parameters. RMSE values are shown for each molecule and averaged over all states

	SA-CASSCF	XS-CASSCF [0.0,0.5]	XS-CASSCF opt.
Ethene	0.96	0.63	0.27, [0.4,—]
Butadiene	1.07	0.36	0.32, [0.1,0.7]
Hexatriene	1.01	0.18	0.03, [0.0,0.0]
Octatetraene	0.97	0.27	0.12, [0.0,0.0]
Naphthalene	0.76	0.46	0.41, [0.0,0.7]
All states	0.96	0.38	0.27

Applying XS-CASSCF_[*µ*,*ν*]_ using molecule-specific *µ* and *ν* parameters further reduces the RMSE. However, the reduction is not as dramatic. The overall RMSE is reduced by about another tenth of an eV to 0.27 eV. The improvements of the individual molecules are no more than 0.15 eV except for the case of ethene where a reduction from 0.63 to 0.27 eV is observed. Note, however, that ethene is somewhat an outlier case as the smallest molecule considered here. Note also that ethene possesses only one CSF in the expansion space and is, hence, unaffected by *ν*.

It is interesting to note from Table 4 that the optimal *µ* parameter is usually close to zero. A value of *µ* = 0.0 means that one simply has to remove the exchange integral *g*_*pq*,*qp*_ in eqn (6) to obtain the S_1_ energy. The S_1_ energy is then entirely determined by the one-electron terms and the Coulomb integral but independent of the exchange integral. As a consequence, the singlet–triplet gap is then only equal to one time the exchange integral rather than twice as large, as one would expect from eqn (8). This finding aligns with discussions by Becke who arrived at an analogous conclusion *via* the adiabatic connection and virial theorem.^54,78^

The discussion of optimal *ν*-values is a bit more subtle. The *ν*-values only affect states that involve significant mixing between several configurations. For butadiene and naphthalene, where higher energy multiconfigurational covalent states are involved in the dataset, we find an optimal value of *ν* = 0.7. By contrast, in the cases of hexatriene and octatetraene, where no such states were included in the QUESTDB reference the optimal value obtained is *ν* = 0.0. We conclude that in some cases the combination [*µ*,*ν*] = [0.0,0.0] yields extremely accurate results, especially when only the HOMO/LUMO transition is of interest. Conversely, if higher energy multiconfigurational covalent states, such as the 1^1^B_u_^−^ state of hexatriene (see Table 2) are of interest, then a higher value of *ν* (0.5 or 0.7) is the safer choice. As shown in the middle column of Table 4 the combination of [0.0, 0.5] indeed provides good results over all types of states and we suggest this as a default starting point for XS-CASSCF.

Next, we turn the focus to various molecules with heteroatoms leading to a more challenging set of nπ* and ππ* states. Table 5 presents the RMSE values in analogy to the previous discussion. As before, the standard SA-CASSCF method is insufficient to provide accurate excitation energies. All individual RMSEs are above 0.80 eV, the only exception being cyanoformaldehyde with a slightly lower RMSE (0.70 eV). The RMSE considering all states is 0.90 eV, similar to the hydrocarbons.

**Table 5:** RMSE values (eV) for the heteroatom test set calculated using standard SA-CASSCF, XS-CASSCF with globally and individually optimized parameters. RMSE values are shown for each molecule and averaged over all states

Molecule	SA-CASSCF	XS-CASSCF [0.0,0.5]	XS-CASSCF opt.
Pyridine	0.89	0.54	0.54, [0.0,0.4]
Pyrimidine	0.86	0.72	0.72, [0.0,0.5]
Triazine	0.84	0.61	0.60, [0.0,0.6]
Acrolein	1.00	0.85	0.80, [0.4,0.4]
Acrolein (^1^A′)^a^	1.38	0.64	0.57, [0.1,0.9]
Cyanoformaldehyde	0.70	0.74	0.70, [0.4,1.0]
Cyclopentadienone	0.96	0.56	0.56, [0.0,0.6]
RMSE for all states	0.90	0.67	0.66

a Using only the three ^1^A′ states for the state averaging.

Applying the globally optimized XS-CASSCF_[0.0,0.5]_ method leads to improvements for most molecules but clearly not as pronounced as in the case of the hydrocarbons. Fairly substantial improvements are observed for only cyclopentadienone and pyridine, with more minor improvements for the other molecules. In the case of cyanoformaldehyde there is even a slight increase to 0.74 eV. Nonetheless, the overall RMSE is slightly improved to 0.67 eV. Interestingly, using molecule-specific optimized *µ*/*ν* parameters provides almost no improvement on the [0.0,0.5] results.

Before moving on, we want to revisit acrolein, the molecule with the largest XS-CASSCF RMSE in Table 5. The original computation considering state-averaging over six excited states along with the ground state produced an RMSE of 1.00 eV for standard SA-CASSCF with only marginal reductions (0.85 and 0.80 eV) for the XS-CASSCF variants. For comparison we did computations where the state averaging is done over only the three ^1^A′ states, that is, the ground state and two ππ* excited states, and the results are also listed in Table 5. In this case, the utility of the XS-CASSCF method is much more immediately apparent. The original RMSE for the SA-CASSCF method is equal to 1.38 eV, which is too large for most practical applications. The error is reduced dramatically to 0.64 eV for [*µ*,*ν*] equal to [0.0,0.5] and to 0.57 eV for [*µ*,*ν*] equal to [0.1,0.9]. The difficulties for the original procedure can be explained *via* contributions to the ground state by a configuration that contains two singly occupied orbitals that are singlet coupled and is, therefore, targeted by the shift operator in eqn (16).

**Fig. 8:**
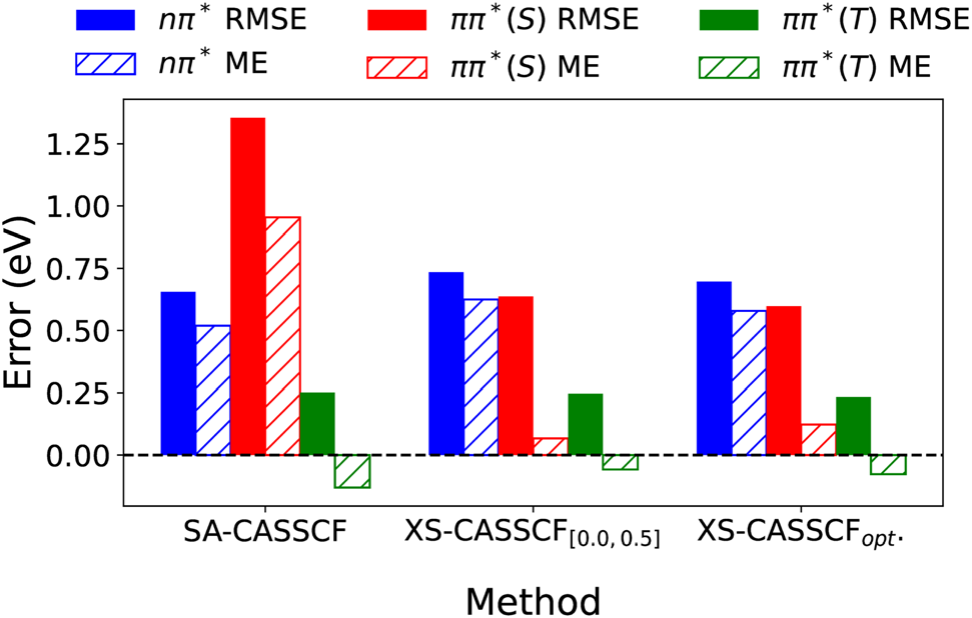
Root mean squared error (RMSE) and mean error (ME) considering the nπ* (blue), singlet ππ* (red) and triplet ππ* (green) excited states for all molecules in the set.

Next, we were interested in studying our results from a somewhat different perspective. The excited states of all molecules were split into three subsets – nπ*, ππ* (singlet, S) and ππ* (triplet, T) – and we computed RMSE and ME for each subset, as shown in Fig. 8. This figure illustrates the dramatic improvement obtained for ππ* singlet excited states where the RMSE drops from 1.35 eV for SA-CASSCF to 0.64 eV for XS-CASSCF_[0.0,0.5]_ and further to 0.60 eV for individually optimized *µ* and *ν* values (XS-CASSCF_opt_). The change in ME is even more dramatic dropping from 0.95 eV to 0.07 and 0.16 eV highlighting that the systematic overestimation of ππ* energies is almost completely eliminated.

It is also important to highlight that RMSE and ME values for the nπ* and triplet ππ* excitation energies are almost unaltered. Per design, XS-CASSCF only affects the singlet ππ* states leaving the others unaffected. The small variations that are observed, mostly derive from the changes in the state-averaged molecular orbitals used rather than from the direct influence of the scaled coupling terms.

**Fig. 9:**
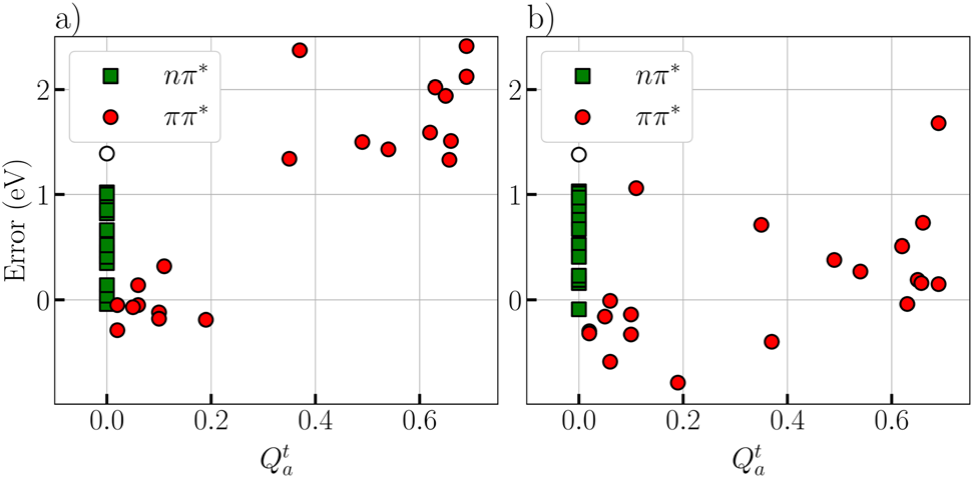
Distribution of signed errors (eV) relative to the TBE values for all singlet states in the dataset: (a) standard CASSCF and (b) XS-CASSCF using optimized *µ* and *ν* values for each molecule. States are grouped by type: ππ* (filled circles) and nπ* (squares); the out-of-plane ππ* state of cyanoformaldehyde is shown as an empty circle.

Finally, we wanted to evaluate the impact of the XS-CASSCF method on states of varying degree of ionic character as determined by the *Q*^t^_a_ diagnostic. For this purpose, we plot the errors for each state *vs.* the *Q*^t^_a_ diagnostic considering standard SA-CASSCF and individually optimized XS-CASSCF, see Fig. 9. As discussed previously,^36^ the *Q*^t^_a_ diagnostic is effective in predicting errors in SA-CASSCF; indeed all states with *Q*^t^_a_ above 0.3 show errors above 1 eV and some of these even above 2 eV. Applying XS-CASSCF now moves all these states (except for one) well into the ±1 eV error range highlighting the greatly improved description. The exception is acrolein with an error of 1.7 eV but, as discussed above, acrolein is a somewhat pathological case and, indeed, the error of this state is reduced to 0.27 eV when state averaging is performed over only the three ^1^A′ states (see discussion above). Thus, in summary, XS-CASSCF is shown to be very effective at lowering the energies of the ionic states into the right place.

While XS-CASSCF has a strong impact on the ionic ππ* states, its effect on the other states is much reduced. Starting with the nπ* states, shown as green squares in Fig. 9, we find that these are very much unaltered by the procedure. Similarly, triplet states (not shown in Fig. 9) are almost unaffected. The covalent ππ* states, represented by the 9 red dots with *Q*^t^_a_ values below 0.3 exhibit errors in the range from −0.5 to +0.5 eV in the standard SA-CASSCF calculations. The energy of these states is generally lowered by XS-CASSCF but, clearly, the lowering is much less pronounced than in the case of the ionic states highlighting the power of XS-CASSCF to single out the ionic states. Using XS-CASSCF seven covalent states yield errors in the range from −0.6 to 0.0 eV, and only two states yield errors out of this range, the states 3^1^A′ for acrolein (−0.79 eV) and 3^1^A_g_ for cyclopentadienone (1.06 eV).

Finally, it is of interest to compare the results with time-dependent density functional theory. Using the popular CAM-B3LYP/aug-cc-pVDZ method as an example, we get an RMSE of 0.65 eV over all states, which is similar but slightly worse when compared to XS-CASSCF_[0,0.05]_ (0.59 eV). Note, moreover, that XS-CASSCF is in many ways significantly more robust than TDDFT or any single reference method, allowing the treatment of doubly excited states and open-shell ground states. Crucially, TDDFT is completely unable to describe the pQDM twisting (Fig. 7, see also ref. 79), and is problematic in the description of S_1_/T_1_ gaps as discussed in the next section.

### 4.4 Outlook: molecular materials

**Fig. 10:**
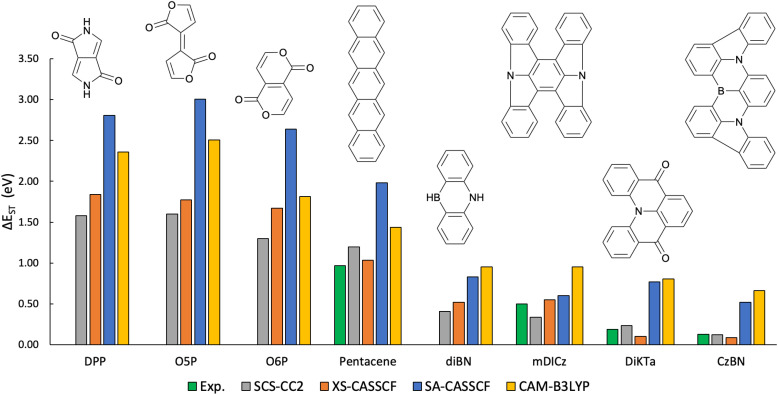
Comparison of the singlet–triplet energy gaps (Δ*E*_ST_ in eV) for a series of chromophores relevant to modern organic optoelectronics. Molecules on the left (DPP, O5P, O6P, and pentacene) are characterized by wide gaps relevant to singlet fission, while those on the right (diBN, mDICz, DiKTa, and CzBN) represent narrow-gap multiresonant emitters. Computational results obtained with XS-CASSCF (orange) are compared against experimental values (green), SCS-CC2 benchmarks (gray), standard SA-CASSCF (blue), and TDDFT/CAM-B3LYP (yellow).

Having presented detailed and systematic results on a variety of model systems, we are now interested in illustrating the performance of XS-CASSCF on a set of more realistic molecular materials. For this purpose, we choose a set of molecules with varying S_1_/T_1_ gaps ranging from about 0.1 eV to more than 1.5 eV. These molecules are illustrated in Fig. 10. To the left, we present diketopyrrolopyrrole (DPP),^80,81^ the two Pechmann dye cores O5P and O6P,^3,82^ and pentacene, all of which possess particularly wide S_1_/T_1_ gaps and are therefore of interest for singlet fission photovoltaics.^1,76^ To the right, we present the two multiresonant emitters DiKTa^83^ and CzBN,^84^ which are noteworthy for their particularly narrow S_1_/T_1_ gaps. We also include dibenzo-azaborine (diBN), a building block for CzBN and other multiresonant emitters, and mDICz, the emitter in a recently developed matrix-free hyperfluorescent OLED,^5^ both of which possess mid-range S_1_/T_1_ gaps.

From a computational point of view, it is noteworthy that the molecules on both sides of Fig. 10 pose significant challenges to current quantum chemistry methods. Modeling the singlet fission process requires the inclusion of doubly excited states to include the triplet pair state,^1,17^ which is not possible with standard single reference methods. MR-TADF materials, on the other hand, pose particular challenges^85,86^ as it is extremely difficult to model their S_1_/T_1_ gaps accurately, with standard TDDFT methods significantly overshooting their values.

In view of the importance of these materials, we now discuss our results. Fig. 10 presents experimental results along with the calculated S_1_/T_1_ energy gaps (Δ*E*_ST_) obtained using the SCS-CC2, XS-CASSCF, SA-CASSCF, and TDDFT (CAM-B3LYP) methods. Experimental reference values (green) are available for four of the molecules studied (pentacene, mDICz, DiKTa, and CzBN). Otherwise, we consider SCS-CC2 (gray) as an accurate, albeit computationally costly, reference method. Viewing the figure as a whole, the performance of XS-CASSCF (orange) is highly encouraging, mirroring the experimental and SCS-CC2 references across the entire series of molecules. By contrast, standard CASSCF significantly overestimates the S_1_/T_1_ gaps in all cases, in line with the previous discussion. Similarly, we find that TDDFT/CAM-B3LYP overestimates the S_1_/T_1_ gaps, and this becomes particularly pronounced for the narrow-gap systems on the right, where the TDDFT results are off by a factor of three or four. Indeed, the inability of TDDFT to describe multiresonant molecules has been thoroughly discussed in the literature,^85,86^ and we believe that XS-CASSCF may provide a suitable alternative for describing such systems. In summary, XS-CASSCF emerges as a reliable tool for studying a range of modern organic optoelectronic materials.

## 5 Conclusion

In this study, we introduce a multireference electronic structure method, denoted XS-CASSCF, designed to provide improved excitation energies for singlet ionic ππ* states leaving all other types of states largely unaffected. XS-CASSCF is designed as a minimal adjustment to the original CASSCF method, scaling selected Hamiltonian matrix elements based on two adjustable parameters *µ* and *ν* (XS-CASSCF_[*µ*,*ν*]_). We highlighted that the globally optimal parameter combination [*µ*,*ν*] = [0.0,0.5] provided improvements over a range of molecules with different types of excited states and that further improvements were possible using molecule-specific tuning of these parameters. The performance of XS-CASSCF was illustrated using hexatriene and pQDM as detailed test cases before proceeding to a test set considering various π-conjugated molecules and concluding with computations on more realistic modern materials.

For the study of the hexatriene molecule, XS-CASSCF_[0.0,0.0]_ was able to correct the ordering of the states, agreeing with high-quality reference data. The error in the excitation energy for the ionic singlet state was significantly reduced from 2.02 to 0.05 eV; indeed, all four lowest excited states were within 0.05 eV of the reference. We also highlighted that an excellent description up to the fifth excited state was possible when slightly changing the parameters and using XS-CASSCF_[0.0,0.5]_. Concerning the pQDM case, the XS-CASSCF method successfully reproduces the proximity of the states 1^3^A_g_, 1^1^A_u_ and 2^1^A_g_ observed with the MS-CASPT2 method, reducing the error for the ionic state from 2.04 eV (SA-CASSCF) to 0.04 eV (XS-CASSCF_[0.0,0.0]_). The potential energy curves for the twisted structures of this molecule also agreed well with the reference results, highlighting that the method is robust with respect to changes in molecular geometry.

Proceeding to the dataset studied, we found an excellent performance of XS-CASSCF in the case of the hydrocarbons. In this case, the error was reduced by about three quarters (from 0.96 eV to 0.27 eV) when using individually optimized parameters and still by almost two thirds (from 0.96 eV to 0.38 eV) for the globally optimized XS-CASSCF_[0.0,0.5]_ method. Considering the second, more challenging, half of our dataset containing π-conjugated molecules with heteroatoms with a variety of nπ* and ππ* states, more caution is warranted. In this case a significant, but less spectacular, decrease in the average error from 0.90 eV to 0.66 eV was observed obtaining very similar results for XS-CASSCF_[0.0,0.5]_ and its individually optimized variant. While it is encouraging that XS-CASSCF also improves the results in these cases, it should be noted that they illustrate a limitation of the method: if a large number of states of different character are included in the state averaging procedure, then XS-CASSCF can certainly not correct all of them being a method specifically designed to target ionic ππ* states.

Further analysis of the data highlighted that XS-CASSCF produces a substantial improvement for singlet ππ* states. At the same time it leaves nπ* states and triplets largely unaffected. Comparison with our recently developed *Q*^t^_a_ diagnostic^36^ shows that XS-CASSCF correctly targets the ionic states marked by large (>0.3) *Q*^t^_a_ values.

Finally, the XS-CASSCF method was tested on a set of eight realistic molecular materials, ranging from singlet fission candidates to multiresonant emitters. In these systems, XS-CASSCF effectively addressed the systematic overestimation of S_1_/T_1_ gaps inherent to standard SA-CASSCF. At the same time this method provided a robust alternative to TDDFT, which was found to significantly overshoot the excitation energies in narrow-gap multiresonant systems. By mirroring the trends observed in both experimental data and high-level SCS-CC2 benchmarks, XS-CASSCF proved its capability to handle complex, modern organic optoelectronic materials where single-reference methods are often insufficient.

Viewing the above results, we certainly do not claim that XS-CASSCF is a completely blackbox excited state method that can be applied without limit to any class of problem. The presented results, however, suggest that XS-CASSCF can provide very favorable results for a substantial class of molecules providing a way to tackle notoriously challenging excited state computations on ionic ππ* states. As such we believe that XS-CASSCF will be an important addition to the quantum chemistry toolbox. A typical envisaged use case is a wider exploration of the potential energy surface or dynamics simulations using XS-CASSCF after its accuracy has been verified against a higher-level method on a few selected geometries. Following the results of this study, we suggest XS-CASSCF_[0.0,0.5]_ as the most flexible starting point for these cases. If only a smaller number of states are of interest, XS-CASSCF_[0.0,0.0]_ may be investigated. Further tuning of the parameters may be carried out if necessary. We also note in this context that XS-CASSCF is designed to work with π/n/π* active spaces. We do not suggest including σ or σ* orbitals into the active space as this may lead to double counting of correlation.

The conceptual simplicity of the XS-CASSCF method also makes it directly amenable for additional method developments. XS-CASSCF not only serves as a convenient starting point by providing MOs for further correlated treatment, but the same scaling procedure may also be directly integrated into higher-level methods. Current developments by some of us are concerned with including similar shift parameters into the MRCI method. Similarly, one may consider the XS-CASSCF Hamiltonian as an alternative zero-order Hamiltonian for multireference perturbation theories. The fully self-consistent nature of XS-CASSCF also provides the basis for the implementation of gradients or nonadiabatic couplings and this work is currently planned. In summary, we believe that XS-CASSCF is a promising new addition to the excited state quantum chemistry toolbox tackling a long-known notorious problem in a very targeted fashion.

## Author contributions

Conceptualization: F. P.; formal analysis: F. P., R. S., H. L.; investigation: R. F. K. S., F. P., S. A. d. M., R. L. R. A., L. B.; methodology – R. F. K. S., F. P.; software – R. F. K. S., F. P.; validation – R. F. K. S., S. A. d. M., R. L. R. A.; writing – original draft: R. F. K. S, S. A. d. M., F. P.; writing – review and editing – R. F. K. S., F. P., H. L., R. S.

## Conflicts of interest

There are no conflicts to declare.

## Supplementary Material

SC-017-D5SC09498D-s001

## Data Availability

Data for this article, including Columbus input/output files for all computations performed, are available at Loughborough University's institutional repository at https://doi.org/10.17028/rd.lboro.30665393. Supplementary information (SI): details on parameters (*µ* and *ν* values) employed; excitation energies for pQDM for various *µ*/*ν*; detailed error analysis for all molecules for varying *µ*/*ν*; vertical excitation energies for molecular materials test set. See DOI: https://doi.org/10.1039/d5sc09498d.
