# Supplementary material for: Accurate energies for ππ* excited states via exchange scaling: the XS-CASSCF method
Source: Chem Sci. 2026 May 13;17(26):13186–200. doi: 10.1039/d5sc09498d (PMC13216890; doi:10.1039/d5sc09498d)
Supplement: SC-017-D5SC09498D-s001 [file SC-017-D5SC09498D-s001.pdf]

Supplementary Information (SI) for Chemical Science.
This journal is © The Royal Society of Chemistry 2026

Supporting Information for "Accurate Excitation Energies for $\pi\pi^*$ Excited States via Exchange Scaling: the XS-CASSCF method"

Rene F. K. Spada, Rodolpho L. R. Alves, Sayan Ghosh, Silmar A. do Monte, Lachlan Belcher, Ron Shepard, Hans Lischka, Felix Plasser

(Dated: April 13, 2026)

I. SUPPORTING INFORMATION

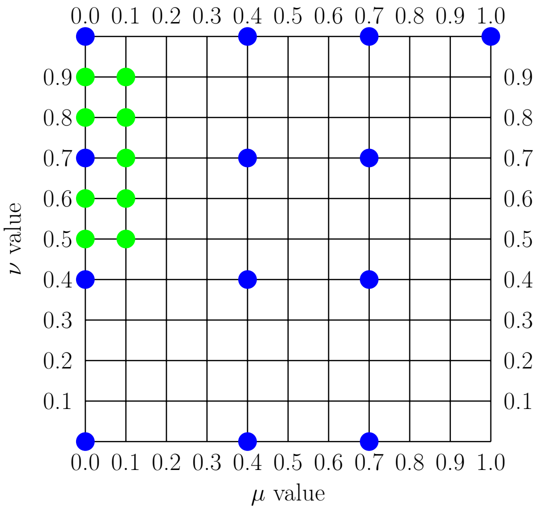

FIG. S1. Grid of μ and ν values employed for the calculations. The blue dots define a sparcer grid that spans the whole intervals and the green dots define a finer grid in a region of interest.

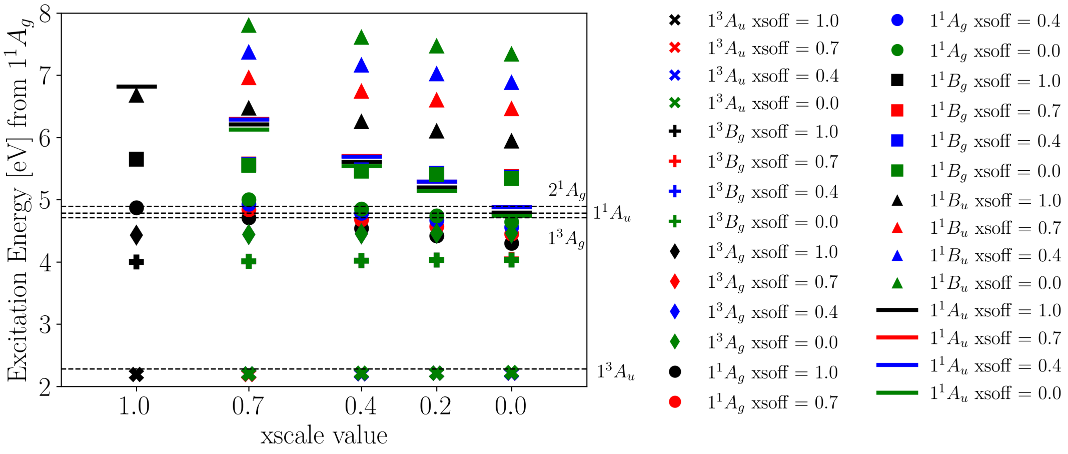

FIG. S2. Vertical excitation energies for the pQDM molecule, for several pairs of μ/ν values. The dashed lines consider the reference CASPT2 values.

TABLE S1. Mean absolute error (MAE), mean error (ME) and root mean squared error (RMSE) for the ethene molecule, considering all states and considering only the singlet states.

μ value	0.0	0.1	0.4	0.7	1.0
MAE for all states	0.585	0.455	0.250	0.555	0.805
MAE only for singlet states	0.810	0.560	0.160	0.790	1.330
ME for all states	-0.585	-0.455	-0.090	0.235	0.525
ME only for singlet states	-0.810	-0.560	0.160	0.790	1.330
RMSE for all states	0.627	0.467	0.266	0.603	0.961
RMSE only for singlet states	0.810	0.560	0.160	0.790	1.330

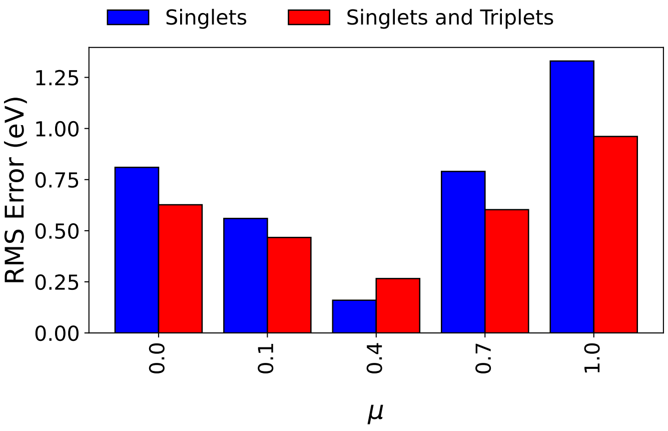

FIG. S3. Root mean squared error (RMSE) for the ethene molecule, considering all states and considering only the singlet states.

TABLE S2. Mean absolute error (MAE), mean error (ME) and root mean squared error (RMSE) for the acrolein molecule, considering all states and considering only the singlet states.

μ value	0.0								0.1				
ν value	0.0	0.4	0.5	0.6	0.7	0.8	0.9	1.0	0.5	0.6	0.7	0.8	0.9
MAE for all states	0.760	0.753	0.753	0.758	0.758	0.760	0.763	0.768	0.707	0.708	0.712	0.713	0.717
MAE only for singlet states	0.925	0.905	0.905	0.907	0.905	0.905	0.905	0.907	0.870	0.870	0.870	0.870	0.873
ME for all states	0.330	0.370	0.380	0.392	0.402	0.413	0.427	0.438	0.363	0.372	0.385	0.397	0.407
ME only for singlet states	0.280	0.330	0.345	0.357	0.370	0.385	0.400	0.412	0.355	0.365	0.380	0.395	0.407
RMSE for all states	0.870	0.851	0.849	0.851	0.851	0.851	0.852	0.855	0.824	0.824	0.827	0.828	0.828
RMSE only for singlet states	1.017	0.989	0.986	0.987	0.986	0.985	0.983	0.985	0.970	0.969	0.971	0.970	0.971

μ value	0.4				0.7				1.0
ν value	0.0	0.4	0.7	1.0	0.0	0.4	0.7	1.0	1.0
MAE for all states	0.587	0.585	0.590	0.602	0.527	0.523	0.527	0.532	0.533
MAE only for singlet states	0.797	0.787	0.788	0.793	0.738	0.733	0.737	0.745	0.748
ME for all states	0.280	0.322	0.350	0.385	0.277	0.317	0.343	0.375	0.387
ME only for singlet states	0.337	0.392	0.427	0.467	0.417	0.467	0.502	0.540	0.633
RMSE for all states	0.801	0.799	0.805	0.816	0.836	0.848	0.862	0.879	1.001
RMSE only for singlet states	0.971	0.967	0.973	0.984	1.021	1.036	1.054	1.074	1.222

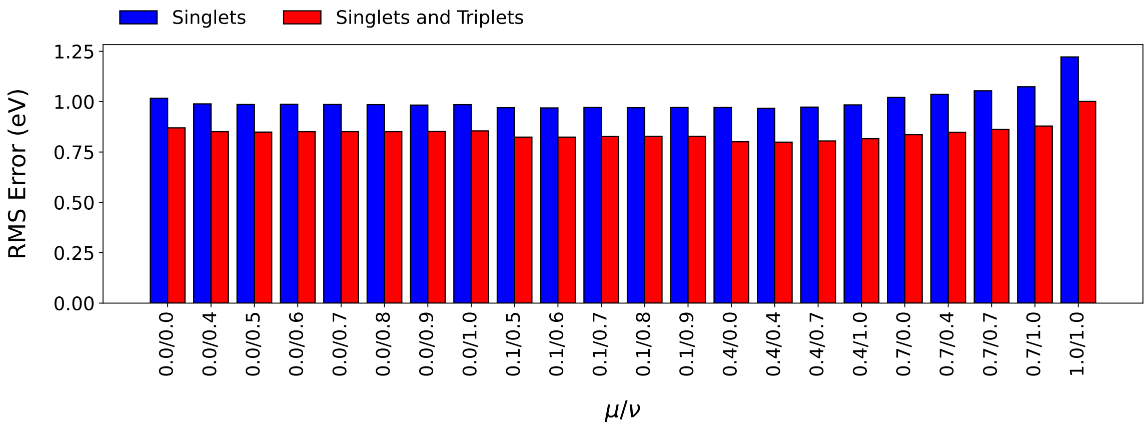

FIG. S4. Root mean squared error (RMSE) for the acrolein molecule, considering all states and considering only the singlet states.

TABLE S3. Mean absolute error (MAE), mean error (ME) and root mean squared error (RMSE) for the acrolein molecule, considering only 3 $^1A'$ states for the state averaging.

μ value	0.0								0.1				
ν value	0.0	0.4	0.5	0.6	0.7	0.8	0.9	1.0	0.5	0.6	0.7	0.8	0.9
MAE	0.520	0.470	0.480	0.480	0.475	0.475	0.470	0.475	0.510	0.510	0.515	0.515	0.515
ME	−0.520	−0.430	−0.420	−0.410	−0.405	−0.395	−0.390	−0.385	−0.280	−0.270	−0.265	−0.255	−0.245
RMSE	0.701	0.637	0.638	0.631	0.624	0.618	0.611	0.611	0.582	0.577	0.579	0.575	0.570
μ value	0.4				0.7				1.0				
ν value	0.0	0.4	0.7	1.0	0.0	0.4	0.7	1.0	1.0				
MAE	0.690	0.680	0.680	0.670	0.805	0.820	0.825	0.810	0.975				
ME	0.030	0.080	0.160	0.180	0.445	0.500	0.535	0.610	0.975				
RMSE	0.691	0.685	0.699	0.694	0.920	0.960	0.983	1.014	1.379				

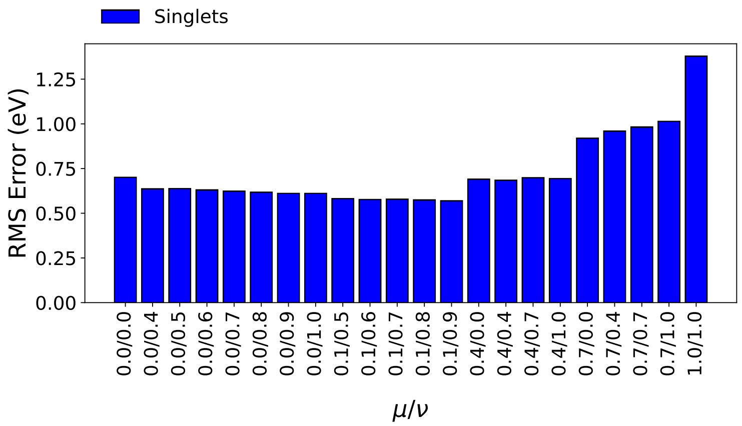

FIG. S5. Root mean squared error (RMSE) for the acrolein molecule, considering only 3 ^1A states for the state averaging.

TABLE S4. Mean absolute error (MAE), mean error (ME) and root mean squared error (RMSE) for the butadiene molecule, considering all states and considering only the singlet states.

μ value	0.0								0.1				
ν value	0.0	0.4	0.5	0.6	0.7	0.8	0.9	1.0	0.5	0.6	0.7	0.8	0.9
MAE for all states	0.305	0.268	0.260	0.255	0.253	0.250	0.247	0.247	0.245	0.247	0.247	0.247	0.250
MAE only for singlet states	0.490	0.410	0.395	0.385	0.380	0.375	0.370	0.370	0.365	0.370	0.370	0.370	0.375
ME for all states	−0.305	−0.268	−0.260	−0.255	−0.253	−0.250	−0.247	−0.247	−0.185	−0.177	−0.172	−0.172	−0.170
ME only for singlet states	−0.490	−0.410	−0.395	−0.385	−0.380	−0.375	−0.370	−0.370	−0.245	−0.230	−0.220	−0.220	−0.215
RMSE for all states	0.405	0.366	0.360	0.354	0.354	0.353	0.353	0.353	0.323	0.321	0.317	0.317	0.318
RMSE only for singlet states	0.559	0.502	0.493	0.485	0.484	0.483	0.483	0.483	0.440	0.436	0.430	0.430	0.432

μ value	0.4				0.7				1.0
ν value	0.0	0.4	0.7	1.0	0.0	0.4	0.7	1.0	1.0
MAE for all states	0.338	0.350	0.355	0.357	0.448	0.455	0.458	0.460	0.635
MAE only for singlet states	0.545	0.565	0.575	0.580	0.760	0.775	0.780	0.785	1.130
ME for all states	−0.002	0.035	0.050	0.057	0.218	0.255	0.272	0.280	0.495
ME only for singlet states	0.125	0.205	0.235	0.250	0.570	0.645	0.680	0.695	1.130
RMSE for all states	0.406	0.436	0.450	0.457	0.679	0.719	0.738	0.748	1.067
RMSE only for singlet states	0.559	0.601	0.621	0.632	0.950	1.008	1.035	1.048	1.502

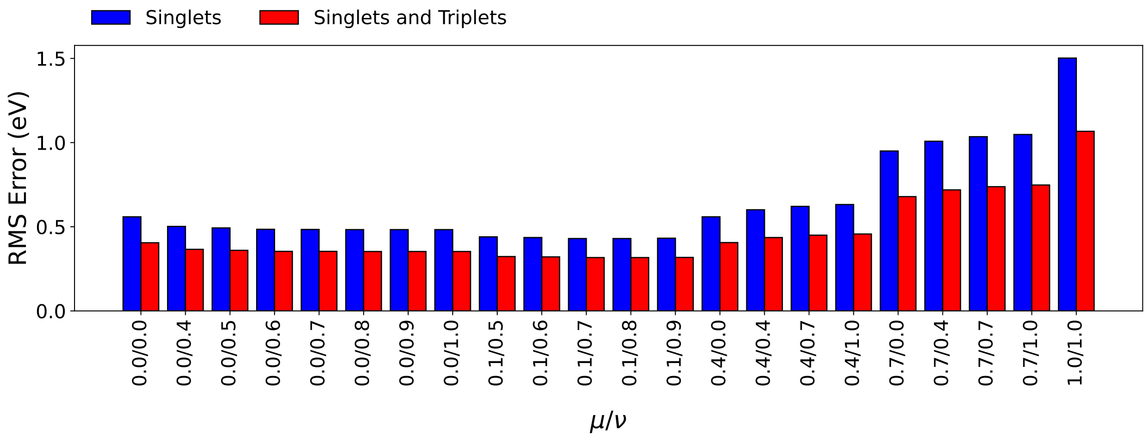

FIG. S6. Root mean squared error (RMSE) for the butadiene molecule, considering all states and considering only the singlet states.

TABLE S5. Mean absolute error (MAE), mean error (ME) and root mean squared error (RMSE) for the cyanoformaldehyde molecule, considering all states and considering only the singlet states.

μ value	0.0								0.1				
ν value	0.0	0.4	0.5	0.6	0.7	0.8	0.9	1.0	0.5	0.6	0.7	0.8	0.9
MAE for all states	0.455	0.460	0.462	0.462	0.460	0.457	0.452	0.447	0.455	0.455	0.455	0.450	0.447
MAE only for singlet states	0.800	0.815	0.815	0.815	0.810	0.805	0.795	0.785	0.800	0.800	0.800	0.790	0.785
ME for all states	0.360	0.365	0.362	0.362	0.360	0.357	0.352	0.342	0.365	0.365	0.360	0.355	0.352
ME only for singlet states	0.620	0.635	0.635	0.635	0.630	0.625	0.615	0.595	0.650	0.650	0.640	0.630	0.625
RMSE for all states	0.723	0.737	0.737	0.737	0.733	0.728	0.718	0.704	0.735	0.735	0.731	0.721	0.716
RMSE only for singlet states	1.012	1.033	1.033	1.033	1.026	1.019	1.005	0.985	1.031	1.031	1.024	1.010	1.003

μ value	0.4				0.7				1.0
ν value	0.0	0.4	0.7	1.0	0.0	0.4	0.7	1.0	1.0
MAE for all states	0.432	0.438	0.438	0.422	0.415	0.425	0.420	0.410	0.412
MAE only for singlet states	0.755	0.765	0.765	0.735	0.720	0.740	0.730	0.710	0.715
ME for all states	0.362	0.367	0.362	0.347	0.365	0.370	0.365	0.350	0.353
ME only for singlet states	0.665	0.685	0.675	0.645	0.700	0.720	0.710	0.680	0.715
RMSE for all states	0.717	0.731	0.726	0.697	0.715	0.734	0.724	0.700	0.700
RMSE only for singlet states	1.006	1.027	1.020	0.978	1.004	1.032	1.018	0.983	0.983

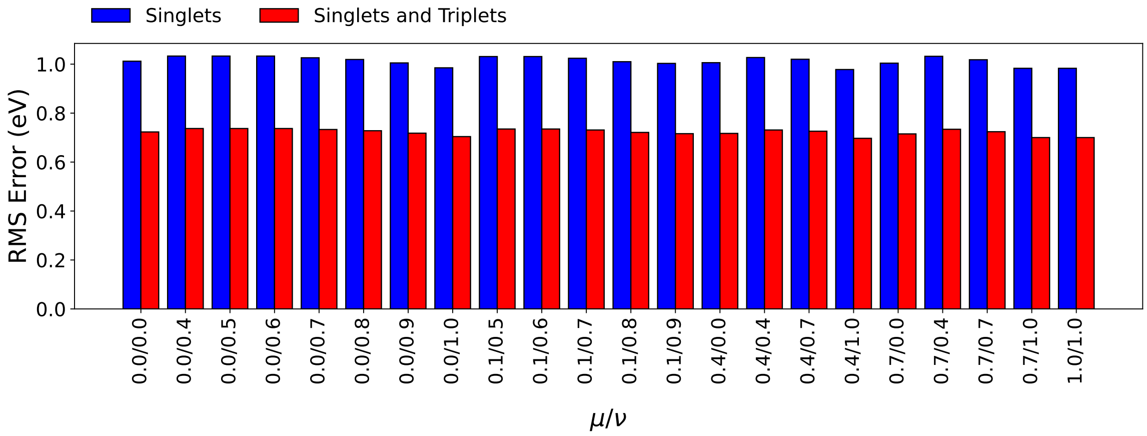

FIG. S7. Root mean squared error (RMSE) for the cyanoformaldehyde molecule, considering all states and considering only the singlet states.

TABLE S6. Mean absolute error (MAE), mean error (ME) and root mean squared error (RMSE) for the cyclopentadienone molecule, considering all states and considering only the singlet states.

μ value	0.0								0.1				
ν value	0.0	0.4	0.5	0.6	0.7	0.8	0.9	1.0	0.5	0.6	0.7	0.8	0.9
MAE for all states	0.459	0.474	0.478	0.482	0.488	0.493	0.501	0.507	0.483	0.487	0.490	0.499	0.504
MAE only for singlet states	0.522	0.550	0.556	0.562	0.572	0.582	0.594	0.604	0.578	0.586	0.592	0.602	0.612
ME for all states	0.446	0.417	0.404	0.393	0.381	0.367	0.352	0.336	0.439	0.427	0.412	0.401	0.387
ME only for singlet states	0.498	0.446	0.424	0.402	0.380	0.354	0.326	0.296	0.498	0.478	0.452	0.426	0.400
RMSE for all states	0.567	0.563	0.562	0.562	0.564	0.567	0.572	0.578	0.597	0.594	0.591	0.592	0.592
RMSE only for singlet states	0.643	0.637	0.635	0.634	0.637	0.641	0.648	0.658	0.696	0.693	0.688	0.687	0.687

μ value	0.4				0.7				1.0
ν value	0.0	0.4	0.7	1.0	0.0	0.4	0.7	1.0	1.0
MAE for all states	0.584	0.551	0.516	0.528	0.677	0.643	0.611	0.568	0.656
MAE only for singlet states	0.776	0.720	0.656	0.678	0.958	0.902	0.844	0.764	0.938
ME for all states	0.580	0.547	0.509	0.466	0.663	0.628	0.596	0.552	0.631
ME only for singlet states	0.776	0.720	0.656	0.574	0.958	0.902	0.844	0.764	0.938
RMSE for all states	0.761	0.737	0.712	0.688	0.920	0.891	0.860	0.820	0.961
RMSE only for singlet states	0.950	0.917	0.882	0.845	1.185	1.145	1.103	1.046	1.249

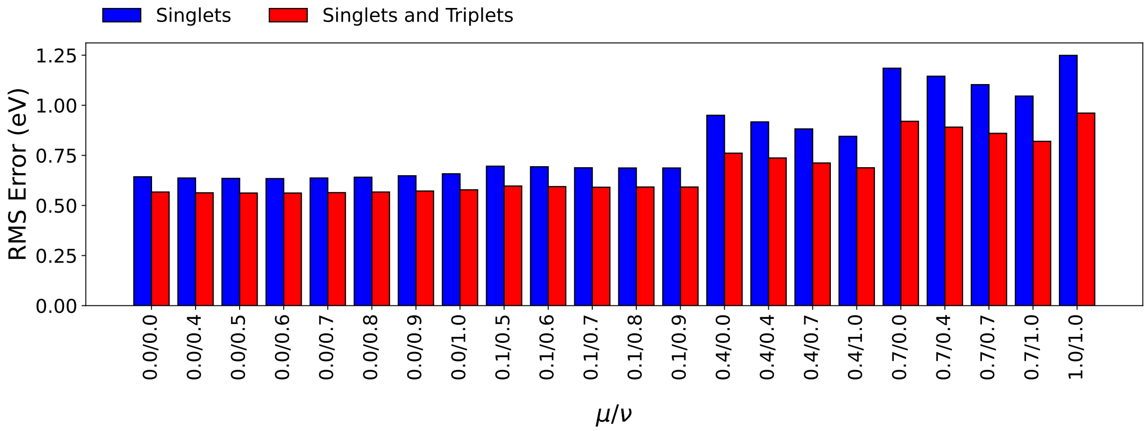

FIG. S8. Root mean squared error (RMSE) for the cyclopentadienone molecule, considering all states and considering only the singlet states.

TABLE S7. Mean absolute error (MAE), mean error (ME) and root mean squared error (RMSE) for the hexatriene molecule, considering all states and considering only the singlet states.

μ value	0.0								0.1				
ν value	0.0	0.4	0.5	0.6	0.7	0.8	0.9	1.0	0.5	0.6	0.7	0.8	0.9
MAE for all states	0.025	0.110	0.133	0.160	0.180	0.200	0.217	0.245	0.165	0.185	0.210	0.230	0.248
MAE only for singlet states	0.025	0.200	0.250	0.300	0.345	0.385	0.425	0.470	0.310	0.355	0.400	0.445	0.480
ME for all states	−0.025	−0.015	−0.023	−0.035	−0.050	−0.070	−0.098	−0.125	0.040	0.025	0.010	−0.010	−0.038
ME only for singlet states	−0.025	−0.010	−0.030	−0.060	−0.095	−0.145	−0.205	−0.270	0.100	0.065	0.030	−0.015	−0.080
RMSE for all states	0.029	0.143	0.179	0.217	0.253	0.291	0.334	0.384	0.231	0.256	0.284	0.315	0.344
RMSE only for singlet states	0.029	0.200	0.252	0.306	0.358	0.411	0.472	0.542	0.326	0.361	0.401	0.445	0.487

μ value	0.4				0.7				1.0
ν value	0.0	0.4	0.7	1.0	0.0	0.4	0.7	1.0	1.0
MAE for all states	0.245	0.258	0.300	0.353	0.422	0.438	0.405	0.443	0.532
MAE only for singlet states	0.450	0.480	0.575	0.690	0.795	0.830	0.770	0.865	1.035
ME for all states	0.205	0.223	0.190	0.118	0.372	0.392	0.365	0.297	0.477
ME only for singlet states	0.450	0.480	0.405	0.240	0.795	0.830	0.770	0.615	0.985
RMSE for all states	0.356	0.461	0.498	0.517	0.632	0.733	0.761	0.751	1.011
RMSE only for singlet states	0.501	0.651	0.703	0.731	0.892	1.036	1.075	1.061	1.429

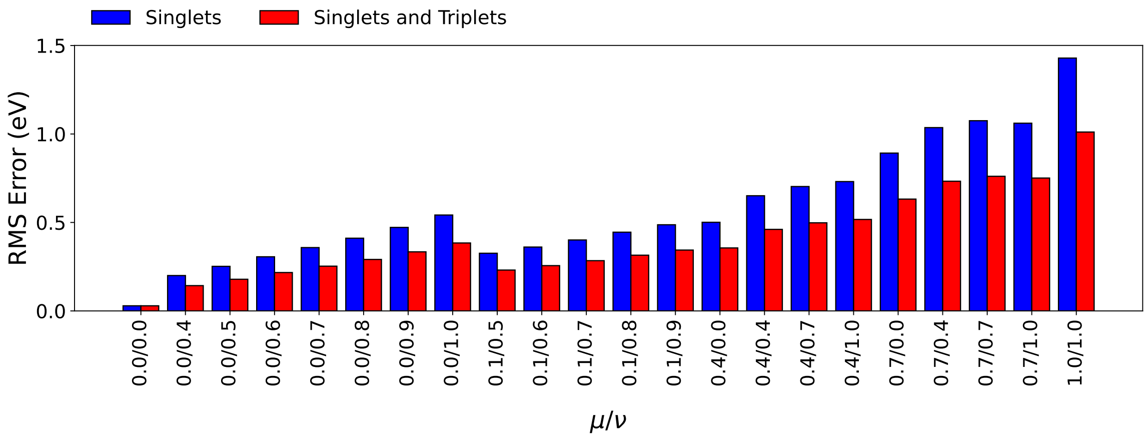

FIG. S9. Root mean squared error (RMSE) for the hexatriene molecule, considering all states and considering only the singlet states.

TABLE S8. Mean absolute error (MAE), mean error (ME) and root mean squared error (RMSE) for the naphthalene molecule, considering all states and considering only the singlet states.

μ value	0.0								0.1				
ν value	0.0	0.4	0.5	0.6	0.7	0.8	0.9	1.0	0.5	0.6	0.7	0.8	0.9
MAE for all states	0.470	0.343	0.290	0.305	0.323	0.345	0.362	0.397	0.343	0.312	0.330	0.350	0.372
MAE only for singlet states	0.815	0.560	0.455	0.490	0.525	0.565	0.600	0.645	0.560	0.500	0.540	0.580	0.620
ME for all states	0.400	0.272	0.220	0.165	0.098	0.025	−0.057	−0.143	0.273	0.212	0.150	0.075	−0.008
ME only for singlet states	0.815	0.560	0.455	0.340	0.205	0.055	−0.110	−0.285	0.560	0.440	0.310	0.160	−0.010
RMSE for all states	0.601	0.495	0.459	0.430	0.408	0.411	0.440	0.510	0.520	0.479	0.448	0.434	0.447
RMSE only for singlet states	0.840	0.688	0.636	0.596	0.564	0.568	0.610	0.705	0.725	0.666	0.623	0.602	0.620

μ value	0.4				0.7				1.0
ν value	0.0	0.4	0.7	1.0	0.0	0.4	0.7	1.0	1.0
MAE for all states	0.662	0.535	0.363	0.422	0.805	0.682	0.508	0.442	1.110
MAE only for singlet states	1.200	0.950	0.600	0.700	1.485	1.240	0.895	0.740	1.110
ME for all states	0.588	0.465	0.292	0.062	0.730	0.608	0.438	0.207	0.638
ME only for singlet states	1.200	0.950	0.600	0.130	1.485	1.240	0.895	0.430	1.077
RMSE for all states	0.873	0.751	0.597	0.514	1.076	0.950	0.777	0.614	0.762
RMSE only for singlet states	1.228	1.056	0.835	0.712	1.516	1.337	1.092	0.856	1.068

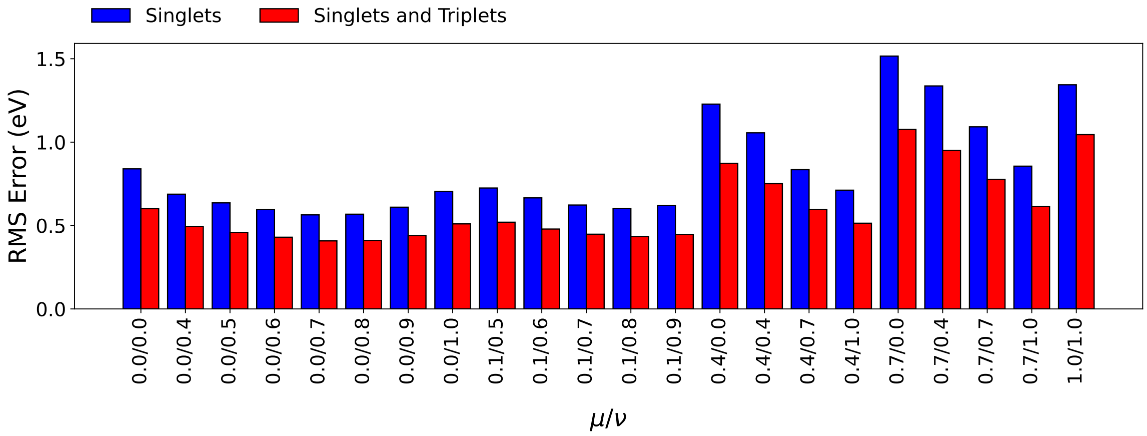

FIG. S10. Root mean squared error (RMSE) for the naphthalene molecule, considering all states and considering only the singlet states.

TABLE S9. Mean absolute error (MAE), mean error (ME) and root mean squared error (RMSE) for the octatetraene molecule, considering all states and considering only the singlet states.

μ value	0.0								0.1				
ν value	0.0	0.4	0.5	0.6	0.7	0.8	0.9	1.0	0.5	0.6	0.7	0.8	0.9
MAE for all states	0.098	0.188	0.205	0.223	0.238	0.255	0.268	0.285	0.230	0.245	0.263	0.275	0.290
MAE only for singlet states	0.175	0.345	0.380	0.410	0.435	0.460	0.480	0.505	0.430	0.460	0.485	0.510	0.530
ME for all states	0.018	0.057	0.055	0.052	0.042	0.030	0.013	−0.010	0.105	0.100	0.092	0.080	0.060
ME only for singlet states	0.015	0.085	0.080	0.070	0.045	0.010	−0.030	−0.085	0.180	0.170	0.145	0.120	0.070
RMSE for all states	0.125	0.252	0.275	0.295	0.311	0.327	0.342	0.365	0.330	0.347	0.359	0.372	0.380
RMSE only for singlet states	0.176	0.355	0.388	0.416	0.437	0.460	0.481	0.512	0.466	0.490	0.506	0.524	0.535

μ value	0.4				0.7				1.0
ν value	0.0	0.4	0.7	1.0	0.0	0.4	0.7	1.0	1.0
MAE for all states	0.213	0.280	0.330	0.370	0.357	0.400	0.403	0.440	0.507
MAE only for singlet states	0.415	0.550	0.640	0.700	0.705	0.785	0.795	0.855	1.005
ME for all states	0.208	0.250	0.235	0.185	0.347	0.390	0.383	0.330	0.472
ME only for singlet states	0.415	0.490	0.450	0.330	0.705	0.785	0.755	0.635	0.935
RMSE for all states	0.396	0.521	0.553	0.548	0.626	0.746	0.775	0.753	0.971
RMSE only for singlet states	0.559	0.737	0.782	0.774	0.885	1.055	1.096	1.065	1.373

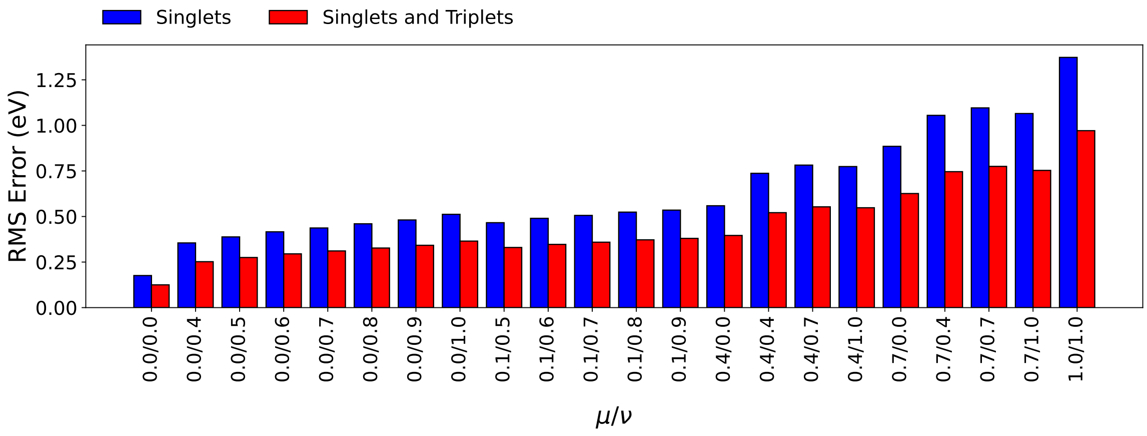

FIG. S11. Root mean squared error (RMSE) for the octatetraene molecule, considering all states and considering only the singlet states.

TABLE S10. Mean absolute error (MAE), mean error (ME) and root mean squared error (RMSE) for the pyridine molecule, considering all states and considering only the singlet states.

μ value	0.0								0.1				
ν value	0.0	0.4	0.5	0.6	0.7	0.8	0.9	1.0	0.5	0.6	0.7	0.8	0.9
MAE for all states	0.484	0.436	0.456	0.474	0.494	0.514	0.532	0.586	0.470	0.490	0.514	0.530	0.550
MAE only for singlet states	0.530	0.468	0.492	0.518	0.542	0.568	0.590	0.657	0.508	0.535	0.565	0.585	0.610
ME for all states	0.364	0.256	0.212	0.166	0.110	0.046	−0.024	−0.098	0.270	0.222	0.170	0.106	0.038
ME only for singlet states	0.530	0.397	0.343	0.283	0.212	0.133	0.045	−0.048	0.417	0.355	0.290	0.210	0.125
RMSE for all states	0.566	0.543	0.545	0.554	0.581	0.626	0.685	0.762	0.565	0.567	0.584	0.609	0.658
RMSE only for singlet states	0.615	0.587	0.589	0.601	0.632	0.684	0.751	0.839	0.611	0.614	0.634	0.663	0.719

μ value	0.4				0.7				1.0
ν value	0.0	0.4	0.7	1.0	0.0	0.4	0.7	1.0	1.0
MAE for all states	0.702	0.608	0.558	0.620	0.842	0.762	0.656	0.670	0.714
MAE only for singlet states	0.792	0.675	0.613	0.690	0.963	0.860	0.728	0.748	0.798
ME for all states	0.566	0.472	0.342	0.148	0.698	0.614	0.508	0.334	0.514
ME only for singlet states	0.792	0.675	0.512	0.270	0.963	0.860	0.728	0.508	0.738
RMSE for all states	0.780	0.710	0.656	0.677	0.943	0.867	0.802	0.744	0.888
RMSE only for singlet states	0.855	0.775	0.713	0.737	1.039	0.951	0.877	0.812	0.974

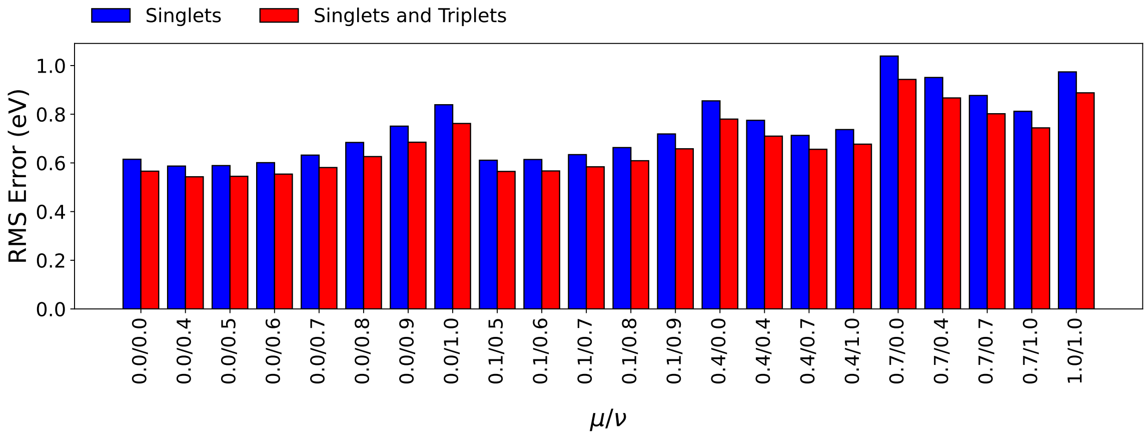

FIG. S12. Root mean squared error (RMSE) for the pyridine molecule, considering all states and considering only the singlet states.

TABLE S11. Mean absolute error (MAE), mean error (ME) and root mean squared error (RMSE) for the pyrimidine molecule, considering all states and considering only the singlet states.

μ value	0.0								0.1				
ν value	0.0	0.4	0.5	0.6	0.7	0.8	0.9	1.0	0.5	0.6	0.7	0.8	0.9
MAE for all states	0.660	0.631	0.651	0.673	0.689	0.697	0.701	0.697	0.661	0.680	0.693	0.706	0.710
MAE only for singlet states	0.713	0.677	0.700	0.725	0.743	0.753	0.758	0.753	0.710	0.732	0.747	0.762	0.767
ME for all states	0.563	0.491	0.454	0.413	0.363	0.303	0.230	0.149	0.499	0.457	0.404	0.346	0.276
ME only for singlet states	0.713	0.633	0.590	0.542	0.483	0.413	0.328	0.233	0.643	0.595	0.533	0.465	0.383
RMSE for all states	0.716	0.716	0.716	0.724	0.737	0.755	0.785	0.824	0.728	0.727	0.730	0.745	0.765
RMSE only for singlet states	0.760	0.759	0.759	0.769	0.782	0.802	0.835	0.878	0.772	0.771	0.774	0.790	0.813

μ value	0.4				0.7				1.0
ν value	0.0	0.4	0.7	1.0	0.0	0.4	0.7	1.0	1.0
MAE for all states	0.827	0.767	0.714	0.731	0.949	0.891	0.779	0.754	0.773
MAE only for singlet states	0.902	0.830	0.768	0.788	1.040	0.972	0.838	0.810	0.828
ME for all states	0.719	0.656	0.534	0.334	0.834	0.774	0.659	0.466	0.596
ME only for singlet states	0.902	0.830	0.688	0.455	1.040	0.972	0.838	0.613	0.768
RMSE for all states	0.854	0.808	0.763	0.758	0.989	0.919	0.846	0.783	0.858
RMSE only for singlet states	0.909	0.858	0.809	0.803	1.055	0.978	0.898	0.828	0.909

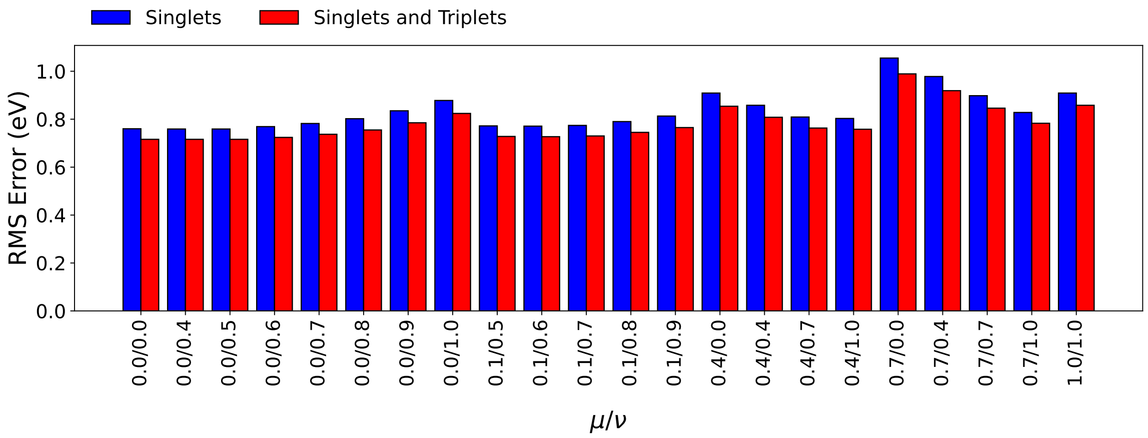

FIG. S13. Root mean squared error (RMSE) for the pyrimidine molecule, considering all states and considering only the singlet states.

TABLE S12. Mean absolute error (MAE), mean error (ME) and root mean squared error (RMSE) for the triazine molecule, considering all states and considering only the singlet states.

μ value	0.0								0.1				
ν value	0.0	0.4	0.5	0.6	0.7	0.8	0.9	1.0	0.5	0.6	0.7	0.8	0.9
MAE for all states	0.616	0.526	0.556	0.566	0.582	0.600	0.618	0.658	0.568	0.588	0.596	0.612	0.628
MAE only for singlet states	0.655	0.542	0.580	0.590	0.610	0.630	0.652	0.703	0.593	0.615	0.625	0.645	0.662
ME for all states	0.432	0.306	0.252	0.170	0.086	−0.004	−0.098	−0.218	0.308	0.236	0.148	0.060	−0.036
ME only for singlet states	0.655	0.497	0.430	0.330	0.225	0.115	−0.003	−0.153	0.502	0.415	0.305	0.195	0.078
RMSE for all states	0.657	0.607	0.606	0.604	0.631	0.683	0.759	0.824	0.626	0.620	0.624	0.660	0.719
RMSE only for singlet states	0.698	0.638	0.637	0.633	0.665	0.725	0.814	0.890	0.659	0.650	0.655	0.698	0.765

μ value	0.4				0.7				1.0
ν value	0.0	0.4	0.7	1.0	0.0	0.4	0.7	1.0	1.0
MAE for all states	0.826	0.708	0.646	0.658	0.980	0.870	0.698	0.696	0.722
MAE only for singlet states	0.907	0.760	0.682	0.698	1.095	0.955	0.745	0.743	0.762
ME for all states	0.626	0.508	0.342	0.038	0.772	0.658	0.494	0.224	0.382
ME only for singlet states	0.907	0.760	0.552	0.173	1.095	0.955	0.745	0.407	0.617
RMSE for all states	0.853	0.745	0.693	0.700	1.030	0.902	0.788	0.733	0.841
RMSE only for singlet states	0.920	0.794	0.733	0.742	1.122	0.974	0.844	0.779	0.898

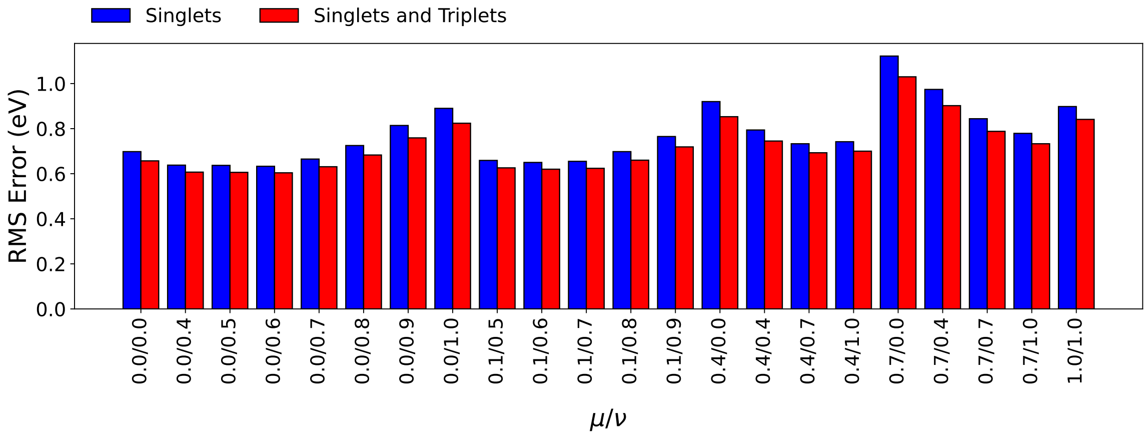

FIG. S14. Root mean squared error (RMSE) for the triazine molecule, considering all states and considering only the singlet states.

TABLE S13. Mean absolute error (MAE), mean error (ME) and root mean squared error (RMSE) for all molecules, considering all states and considering only the singlet states.

μ value	0.0								0.1				
ν value	0.0	0.4	0.5	0.6	0.7	0.8	0.9	1.0	0.5	0.6	0.7	0.8	0.9
MAE for all states	0.470	0.457	0.463	0.474	0.484	0.494	0.502	0.517	0.464	0.472	0.482	0.491	0.501
MAE only for singlet states	0.610	0.586	0.597	0.613	0.628	0.642	0.655	0.676	0.604	0.616	0.630	0.645	0.658
ME for all states	0.269	0.235	0.216	0.194	0.166	0.135	0.100	0.059	0.259	0.236	0.209	0.179	0.143
ME only for singlet states	0.403	0.348	0.318	0.280	0.236	0.186	0.128	0.061	0.394	0.357	0.312	0.262	0.205
RMSE for all states	0.606	0.592	0.590	0.593	0.600	0.614	0.636	0.665	0.598	0.569	0.597	0.606	0.621
RMSE only for singlet states	0.726	0.707	0.705	0.707	0.717	0.735	0.764	0.801	0.718	0.715	0.716	0.727	0.748

μ value	0.4				0.7				1.0
ν value	0.0	0.4	0.7	1.0	0.0	0.4	0.7	1.0	1.0
MAE for all states	0.561	0.525	0.497	0.520	0.665	0.630	0.570	0.561	0.644
MAE only for singlet states	0.770	0.713	0.667	0.700	0.939	0.884	0.789	0.774	0.936
ME for all states	0.429	0.399	0.337	0.235	0.545	0.516	0.459	0.363	0.519
ME only for singlet states	0.686	0.637	0.537	0.370	0.889	0.844	0.752	0.595	0.864
RMSE for all states	0.719	0.691	0.664	0.656	0.869	0.840	0.800	0.762	0.937
RMSE only for singlet states	0.881	0.846	0.809	0.799	1.077	1.039	0.988	0.939	1.154

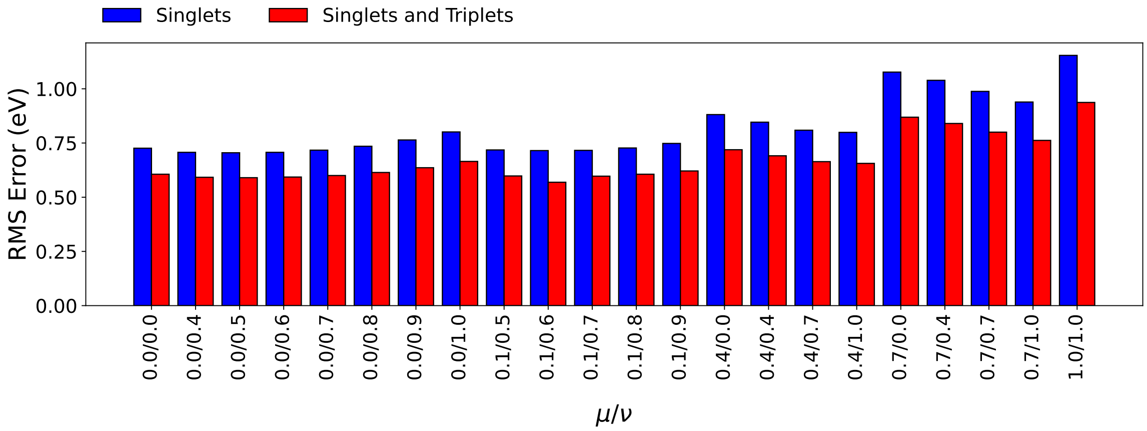

FIG. S15. Root mean squared error (RMSE) for all molecules, considering all states and considering only the singlet states.

TABLE S14. Analysis of the molecular materials test set. Experimental and computed lowest singlet (S_1) and triplet (T_1) energies, and singlet–triplet gaps (ΔE_{ST}) calculated using SCS-CC2, CAM-B3LYP, SA-CASSCF, and XS-CASSCF methods. All energies are given in eV; CAS spaces and μ, ν parameters used in SA-CASSCF and XS-CASSCF are provided.

	Exp.			SCS-CC2			CAM-B3LYP		
	S_1	T_1	ΔE_{ST}	S_1	T_1	ΔE_{ST}	S_1	T_1	ΔE_{ST}
DPP				3.720	2.140	1.580	3.565	1.206	2.359
O5P				3.493	1.891	1.602	3.170	0.663	2.507
O6P				3.958	2.658	1.300	3.706	1.894	1.812
pentacene	1.83	0.86	0.97	2.555	1.358	1.197	2.505	1.607	1.438
diBN				3.757	3.348	0.409	3.915	2.960	0.955
mDICz	2.80	2.30	0.50	3.152	2.812	0.339	3.332	2.380	0.952
DikTa	2.69	2.50	0.19	3.362	3.126	0.236	3.524	2.720	0.804
CzBN	2.52	2.39	0.13	3.147	3.026	0.121	3.395	2.730	0.665

	SA-CASSCF			XS-CASSCF					
	S_1	T_1	ΔE_{ST}	S_1	T_1	ΔE_{ST}	CAS	μ	ν
DPP	4.971	2.161	2.809	4.045	2.208	1.838	(12/10)	0	0.5
O5P	5.080	2.073	3.007	3.870	2.096	1.774	(12/10)	0	0.5
O6P	5.199	2.562	2.637	4.254	2.584	1.670	(12/10)	0	0.5
pentacene	3.583	1.599	1.984	2.632	1.597	1.035	(8/8)	0	1.0
diBN	4.681	3.849	0.833	4.334	3.815	0.519	(8/8)	0	0.5
mDICz	3.992	3.392	0.600	3.951	3.399	0.552	(10/10)	0	0.5
DikTa	4.663	3.892	0.771	4.470	4.370	0.100	(8/8)	0	0.5
CzBN	4.268	3.751	0.518	3.900	3.812	0.089	(10/10)	0	1.0
